# Time to recovery following open and endoscopic carpal tunnel decompression: meta-analysis

**DOI:** 10.1093/bjsopen/zraf085

**Published:** 2025-07-23

**Authors:** Olivia J Hartrick, Rebecca K Turner, Alexander Freethy, Chetan Khatri, Lauren Chong, Ryckie G Wade, Justin C R Wormald, Akira Wiberg, Jeremy N Rodrigues, Conrad Harrison

**Affiliations:** Department of Plastic, Reconstructive, and Hand Surgery, Stoke Mandeville Hospital, Aylesbury, UK; UK Centre for Ecology and Hydrology, Durrell Institute of Conservation and Ecology, University of Kent, Canterbury, UK; Department of Plastic Surgery, Royal Devon University Healthcare NHS Foundation Trust, Exeter, UK; Trauma and Orthopaedic Surgery, University Hospitals Coventry and Warwickshire NHS Trust, Coventry, UK; School of Medicine and Biomedical Sciences, University of Oxford, Oxford, UK; Leeds Institute for Medical Research, University of Leeds, Leeds, UK; Nuffield Department of Orthopaedics, Rheumatology and Musculoskeletal Sciences (NDORMS), University of Oxford, Oxford, UK; Nuffield Department of Orthopaedics, Rheumatology and Musculoskeletal Sciences (NDORMS), University of Oxford, Oxford, UK; Clinical Trials Unit, Warwick Medical School, Warwick, UK; Nuffield Department of Orthopaedics, Rheumatology and Musculoskeletal Sciences (NDORMS), University of Oxford, Oxford, UK

**Keywords:** plastic surgery, orthopaedics

## Abstract

**Background:**

Carpal tunnel release (CTR) can be performed using either an open or endoscopic approach. The patient recovery trajectories remain poorly understood. This study aimed to define and compare patient-reported recovery following unilateral open and endoscopic CTR.

**Methods:**

A PRISMA-compliant, preregistered (CRD42023427718) systematic review was conducted, searching PubMed, Embase, and Cochrane databases on 4 July 2023 and 21 August 2024. Studies were included if they reported recovery data (patient-reported outcome measures (PROMs)) at predefined time points for adults undergoing unilateral CTR. Boston Carpal Tunnel Questionnaire and Quick Disabilities of Arm, Shoulder, and Hand scores were extracted. Standardized mean change (SMC) scores from baseline were pooled using random-effects meta-analysis. An innovative modification of the National Institutes of Health quality assessment tools was used to evaluate the risk of bias.

**Results:**

In all, 49 studies were included (4546 participants included in the analysis; 3137 open CTR, 1409 endoscopic CTR). Both approaches improved PROM scores over 12 weeks, with early (4-week) outcomes strongly correlating (>0.89) with later (12-week) outcomes. Symptoms continued improving up to 104 weeks. At 1 week, open CTR showed symptomatic deterioration (SMC 10.29; 95% confidence interval (c.i.) 6.35 and 14.21 respectively), comparatively, endoscopic CTR demonstrated an improvement (SMC −2.83; 95% c.i. −7.80 and 2.14 respectively). By 2 weeks, symptom severity remained slightly worse in open CTR, but confidence intervals overlapped from week 3 and thereafter open CTR showed greater symptomatic improvement. Most studies had a high risk of bias and measured outcomes too infrequently for a granular comparison.

**Conclusions:**

Patient-reported recovery trajectories for CTR can inform patient counselling and future research. Endoscopic CTR may result in fewer symptoms in the first 2 weeks, but open CTR may offer comparable or potentially greater improvement thereafter. Future trials with high-frequency PROM capture should prioritize early (first 3 weeks) and long-term (≥24 weeks) outcomes.

## Teaser

Recovery trajectories following carpal tunnel release (CTR) remains poorly understood. This study aimed to define and compare patient-reported recovery trajectories after unilateral open and endoscopic CTR. Current evidence suggests that over the first 2 weeks endoscopic CTR may result in less severe postoperative symptoms than open CTR, but greater improvements in symptoms may be seen in open CTR thereafter. Future trials with high-frequency PROM capture should focus on the first 3 weeks after surgery as well as long-term outcomes.

## Introduction

Carpal tunnel release (CTR) is the most common elective hand procedure that surgeons perform in the UK. In England, it is predicted that by the end of 2025 there will be 90 630 CTR operations performed^1^. Despite this high volume, clinicians provide limited and inconsistent information about patients’ expected recovery times. This inconsistency is reflected in the wide range of primary endpoints chosen in CTR studies^2–5^, in the varied follow-up practices of UK hand surgeons^6^, and in the information given to patients^7–9^.

Recently, interest has grown in endoscopic CTR techniques, which may offer faster recovery than the typical open approach. However, this has been met by concerns about potentially higher complication and reoperation rates^10^, additional training requirements, and increased equipment costs^10^. Without a robust understanding of CTR recovery from a patient's perspective, questions cannot be answered about comparative recovery times and the risks and costs of the endoscopic approach cannot be weighed against its potential benefits.

Patient-reported outcome measures (PROMs) quantify symptom severity from the patient's perspective. Researchers commonly use PROM-based questionnaires as primary outcome measures to assess the effectiveness and comparative effectiveness of an intervention. PROMs can also be used longitudinally after surgery to map a patient's recovery over time^11,12^. By analysing time-series data, the rate of a patient's recovery (how quickly their PROM score improves over time) and the time taken to reach their recovered state (when scores begin to plateau) can be understood. This information can then support shared decision-making, help schedule follow-up appointments, and inform both observational and interventional research.

The aim of this study was to map out and compare the recovery trajectories (PROM scores over time) after open and endoscopic CTR through a systematic review and meta-analysis.

## Methods

This study is reported in accordance with the PRISMA statement. The study was preregistered with the systematic review protocol on the PROSPERO database (CRD42023427718).

### Search strategy

This study was designed and executed a search strategy comprising indexed and free terms for the PubMed, Embase, and Cochrane Library databases using the OVID platform. Dates were searched from inception to 21 August 2024. The full search strategy, including terms, filters, and limits, is reported in the *supplementary methods*. An additional manual citation search was performed by one author (O.H.). When full-text articles were difficult to access, assistance was sought from clinical outreach librarians. Trial registries^13^ were searched, and corresponding authors of the included studies were contacted for further information when required.

### Selection process

Rayyan^14^ was used to review and remove duplicate citations. Two authors (O.H., A.F.) independently screened titles and abstracts, and independently assessed full-text papers, with any disagreements about inclusion or exclusion resolved through discussion with a third author (C.H.).

### Eligibility criteria

#### Inclusion criteria

Randomized and non-randomized studies that reported PROM scores for patients at any predefined time point within the first 12 weeks after undergoing unilateral open or endoscopic CTR for carpal tunnel syndrome were included. Studies were only included if full-text manuscripts were accessible.

#### Exclusion criteria

Patients with recurrent carpal tunnel syndrome were excluded. To reduce confounding from frailty and co-morbidities, studies that only included patients aged over 80 years were excluded. In addition, studies where results were reported in a way that prevented comparison with other studies were excluded (for example, if a PROM was scored in a modified manner).

### Data extraction

Two reviewers (O.H., A.F.)independently extracted data from the reports. If study data were not presented in a comparable format (for example, if confidence intervals were presented instead of standard deviations) or there was missing information, the corresponding author was contacted to obtain or confirm data. Up to two attempts were made to contact the corresponding author for additional information if data were missing. If no response was received, and uncertainty persisted, the study was excluded.

Data were extracted from each study arm separately. For example, in comparative studies with a two-arm design (for example, comparing endoscopic CTR with open CTR), each arm was treated as an independent cohort. The number of patients in each arm, mean patient age, sex distribution, intervention type (endoscopic CTR or open CTR), PROMs used, data collection time points, and the mean and standard deviation (s.d.) of PROM scores at each time point were recorded.

### Bias and certainty assessment

In this study, bias was considered as an error that would cause the standardized mean change (SMC) in PROM score at a given time point to be higher or lower than the true population scores. Bias was estimated for each trial arm separately.

The risk of bias was assessed in SMC estimates for each study arm using a modified version of the National Institutes of Health quality assessment tool for all studies^15^. At least two reviewers (O.H., A.F., L.C.) independently assessed the risk of bias for each study in duplicate. The risk of bias score was then discussed for each study, and any differences in scoring were reassessed to reach an agreement. All disagreements were resolved through discussion. A comparative risk of bias tool, such as the Cochrane RoB2 tool, which looks for bias favouring one trial arm over another, was not used because this type of bias was not relevant for plotting recovery trajectories in this study (see *supplementary methods*).

### Effect measures

The most common PROMs reported were the Boston Carpal Tunnel Questionnaire (BCTQ)^11^ and the Quick Disabilities of the Arm, Shoulder and Hand (qDASH)^16^. The BCTQ served as the primary outcome measure for quantitative synthesis. Due to the lack of standardized methods for recording or reporting the visual analogue score for pain, this measure was excluded from the synthesis.

For each study arm, recovery was measured by calculating the SMC^17^ in PROM scores at each available time point. This statistic indicates the amount of symptomatic improvement patients experienced, relative to baseline, at each time point. The SMC score was calculated by subtracting the mean follow-up score from the mean baseline score, dividing it by the baseline s.d. If the pooled s.d. was not reported, either the baseline s.d. or the s.d. at follow-up was used.

### Data synthesis

For each intervention and at each time point, the SMC in BCTQ scores was pooled across studies through random effects meta-analysis. The *I*^2^ statistic was extracted to assess between-study variability. This analysis and the corresponding forest and funnel plots are presented. The Pearson product–moment correlation of BCTQ SMC scores from baseline across time points was calculated for each intervention. Finally, the cumulative change in SMC scores between time points was calculated to evaluate the long-term trajectory of symptom improvement following CTR for each intervention. These trajectories were visualised as point estimates with variance-corrected confidence intervals (see *supplementary methods*). All analyses were conducted in R version 4.4.0^18^, using the metafor^19^ package. The code and the data used in this study are publicly available through GitHub (https://github.com/liv-hartrick/Time-to-recovery-following-open-and-endoscopic-carpal-tunnel-decompression-a-systematic-review).

## Results

### Study selection

**Fig. 1:**
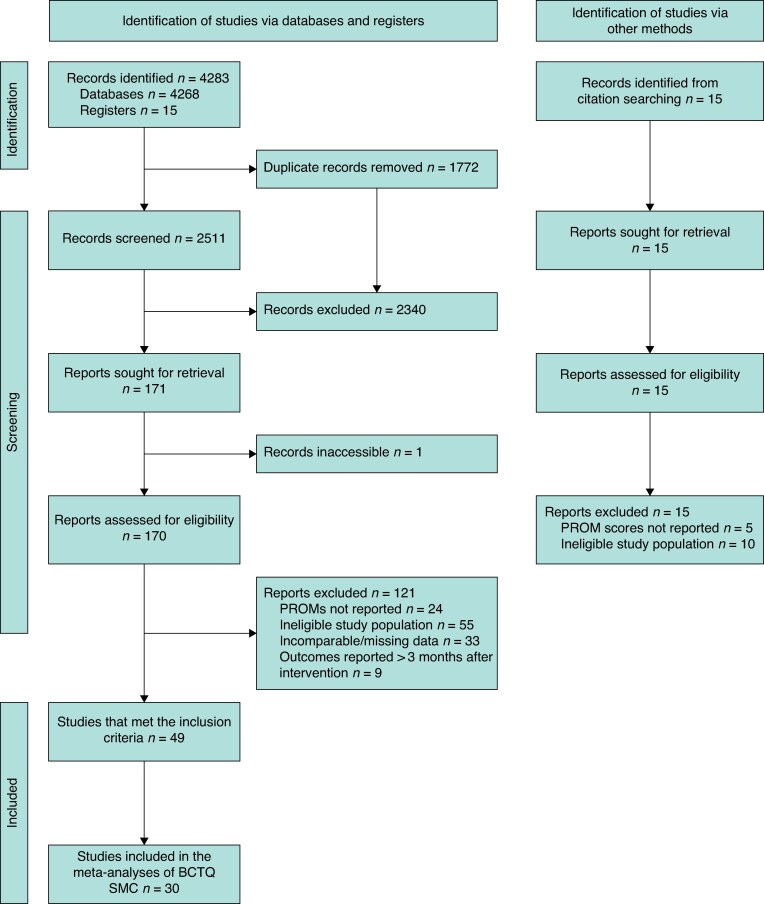
Study selection flow diagram BCTQ, Boston Carpal Tunnel Questionnaire; PROM, patient-reported outcome measure; SMC, standardized mean change.

In all, 2511 unique articles were identified after removing duplicates (*Fig. 1*). Of these, 171 full-text articles were assessed for eligibility and 121 were excluded. Thus, 49 studies that met the inclusion criteria were included in the analysis^12,20–67^.

### Description of study characteristics

*Table S1* summarizes the characteristics of the included studies. Of the 49 studies, 24 were randomized clinical trials (RCTs), 19 were prospective cohort studies, and 6 were retrospective cohort studies. The reported mean age across studies was 52.53 years and the male-to-female ratio was 0.30. In all, 3137 participants underwent open CTR and 1409 participants underwent endoscopic CTR. 573 participants underwent CTR were both open and endoscopic were grouped as one intervention, 196 participants underwent other surgical procedures and 138 participants underwent non-operative interventions.

The choice of PROM varied among the 49 studies. *Table 1* summarizes the frequency of reported PROMs. The BCTQ and qDASH were the most commonly reported PROMs; 30 studies that reported BCTQ scores were included in the meta-analyses.

**Table 1:** Summary of outcome measures reported in the included studies and the frequency of use for each measure

Outcome measure	Frequency
Boston Carpal Tunnel Questionnaire	30
qDASH	11
Michigan Hand Outcomes Questionnaire	5
PROMIS	4
Disabilities of the Arm, Shoulder and Hand	4
Patient Observer Scar Assessment Score	4
Pittsburgh Sleep Quality Index	3
Kelly's grade	3
Other*	8

qDASH, Quick Disabilities of the Arm, Shoulder and Hand; PROMIS, Patient-Reported Outcomes Measurement Information System. *Other patient-reported outcome measures included the 36-Item Short-Form Survey, the 12-Item Short-Form Health Survey, the Insomnia Severity Index, Carpal Tunnel Syndrome Scoring, the McGill Pain Questionnaire, the Global Symptom Score, and the Patient-Specific Functional Scale^68–73^.

### Risk of bias

Four of the 49 studies had a low risk of bias, 17 had a moderate risk of bias, and 28 had a high risk of bias. A summary of the risk of bias for the included studies is provided in *Table S2*.

### Effects of interventions

Data on the effects of interventions are from the studies reporting BCTQ and qDASH scores. Data from 2114 patients and from 77 arms were included in the meta-analyses. The median number of patients per arm was 40 (interquartile range of 28.5–63.0). Studies reporting the BCTQ and qDASH included a total of 634 male and 1480 female participants.

In studies that reported BCTQ or qDASH, there was an overall improvement after both open and endoscopic CTR over time (*Figs S1–S20*). Time series plots illustrate the mean BCTQ score (*Fig. 2*) and qDASH score (*Fig*. *S21*) over time for each study arm.

**Fig. 2:**
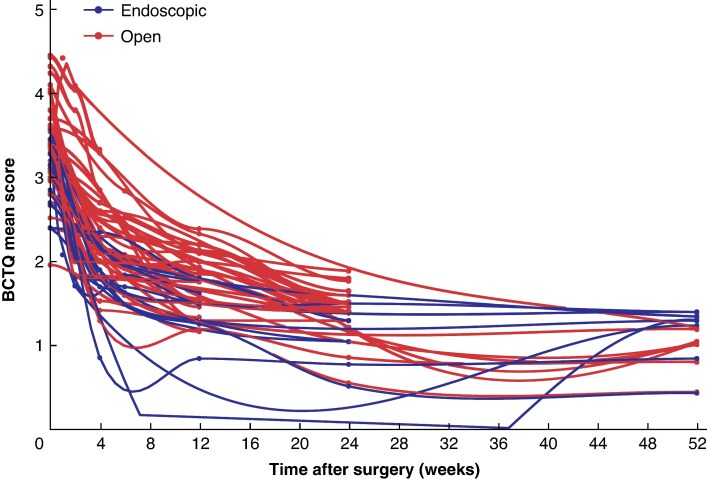
Change in BCTQ score after open and endoscopic carpal tunnel release over time Standardized mean change estimates (points) with smooth interpolation with LOESS function (lines). A decrease in score over time indicates recovery to a plateau. Each line represents a study arm. BCTQ, Boston Carpal Tunnel Questionnaire.

The pooled SMC in BCTQ scores for open and endoscopic CTR arms is summarized in *Table S3*. Forest plots presenting the SMC from baseline of all interventions in studies reporting the BCTQ score are presented in the *supplementary results* for 2, 4, 6, 12, 24, 52, 72, and 104 weeks.

**Fig. 3:**
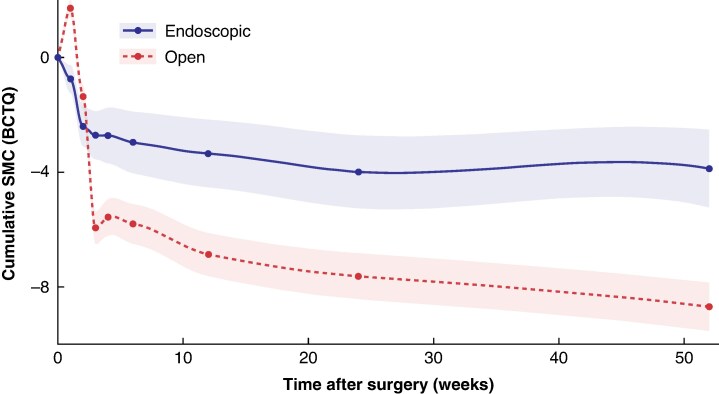
Cumulative SMC in BCTQ scores for endoscopic and open carpal tunnel release In this plot, each point represents the pooled SMC estimate from the 16 different meta-analyses covering weeks 1–52. Not every study will have contributed data to each point. Shaded areas represent 95% confidence intervals. The lower the score, the better the clinical symptoms. The steeper the gradient, the faster the recovery at group level. BCTQ, Boston Carpal Tunnel Questionnaire; SMC, standardized mean change.

At 1 week after the procedure, the SMC in BCTQ score for participants undergoing open CTR was 10.29 (95% confidence interval (c.i.) 6.35 to 14.21; *I*^2^ = 0%). This positive change implies that hand function initially deteriorates. This is not the case for participants undergoing endoscopic CTR, who reported a small improvement (SMC −2.83; 95% c.i. −7.80 to 2.14; *I*^2^ = 0%). At 2 weeks, symptoms are slightly worse for those undergoing open *versus* endoscopic CTR, with an SMC of −2.22 (95% c.i. −4.54 to 0.10; *I*^2^ = 76%) and −3.71 (95% c.i. −7.61 to 0.19; *I*^2^ = 94%), respectively. From 3 weeks, scores for both interventions show sustained improvement, with potentially greater improvements for patients who underwent open CTR (*Fig. 3*).

*Table 2* presents the correlation of mean BCTQ scores across different time points for open and endoscopic CTR. For each intervention, there was a strong correlation (>0.89) between the BCTQ SMC at 4 weeks and that at 12 weeks.

**Table 2:** Correlation of mean Boston Carpal Tunnel Questionnaire scores across different time points

	4 weeks	6 weeks	12 weeks
**Open CTR**			
12 weeks	0.89 (0.71, 0.96)	0.97 (0.85, 0.99)	
24 weeks	0.51 (0.01, 0.80)	0.88 (−0.53, 1.00)	0.57 (0.22, 0.79)
**Endoscopic CTR**			
12 weeks	0.94 (0.68, 0.99)	0.90 (0.31, 0.99)	
24 weeks	0.61 (−0.17, 0.92)		0.75 (0.09, 0.95)

Values in parentheses are 95% confidence intervals. CTR, carpal tunnel release.

## Discussion

This study pools available patient-reported outcomes over time following both open and endoscopic CTR. After the first postoperative week, the SMC analysis suggests that open CTR leads to a worsening of functional and symptomatic scores, whereas endoscopic CTR results in an improvement, as measured by the most frequently used PROM (the BCTQ). After week 3, the SMC analysis suggests that both procedures show similar improvements in BCTQ scores, with open CTR demonstrating the greatest improvement. Scores continue to improve thereafter in both groups for 24 weeks or longer, with potentially greater improvement in the open CTR group. However, because the confidence intervals are approximations, a significant difference cannot be confirmed in the trajectories of recovery following the interventions. Outcomes at 4 weeks are closely correlated with later (12-week) outcomes.

This contextualizes previous studies^3,74–77^ reporting faster recovery of grip and pinch strength, and earlier return to work and daily activities, in patients undergoing endoscopic as opposed to open CTR. Any significant differences, as perceived by patients, are likely to occur in the first 2–3 weeks after surgery. At the group level, there is potentially greater improvement in symptoms following open CTR in the medium term (3–24 weeks), although the clinical and statistical significance of these differences is uncertain. It is plausible, although speculative, that higher reported rates of incomplete release among those undergoing endoscopic CTR^10^ contribute to a comparably lesser improvement in BCTQ scores at the group level, and this should be investigated further.

The findings of the present study should be interpreted with the limitations of the study in mind. First, this meta-analysis should be considered as level 2 evidence, because patients were not all randomized to receive either open or endoscopic CTR. *Figure 2* raises the concern of selection bias, because cohorts receiving open CTR tended to start with higher (poorer) BCTQ scores than those receiving endoscopic CTR. Data for the 1 week postoperative time point were generally limited, with only one study reporting PROM scores for open CTR. This should be considered when interpreting the results of this study. Funnel plots were asymmetric and heterogeneity was high for most time points (*I*^2^ > 90% at 3–24 weeks), potentially a manifestation of publication bias. Further, included studies were generally small, and mostly demonstrated a high risk of bias. Recovery trajectories are pooled at the group level and are likely to differ between individual patients. Future high-quality RCTs are needed to substantiate the findings of the present study and provide health economic analyses.

The information presented here is not only helpful for counselling patients but can inform future prospective study design. Studies aiming to elicit the comparative benefits of the endoscopic approach should aim to capture high-frequency PROM scores over the first 3 weeks after surgery. This may now be possible through advances in PROM capture systems, such as the Ecological Momentary Computerised Adaptive Testing platform, which facilitates high-frequency data capture through low-burden computerized adaptive versions of existing PROMs^78,79^ When determining study duration, it is important to note that patients continue to improve (at the group level) for 24 weeks or longer after their procedure. The correlations provided in this study between early and later outcomes can be used to support efficient and flexible study designs, such as group sequential trials. In group sequential trials, early outcomes are used to model expected later outcomes, while the trial is still recruiting participants. In some cases, early outcome data can then be used to refine sample sizes and facilitate early trial termination^80–83^. Future RCTs in this area should capture reoperation rate, among other adverse events, and secondary subgroup analyses could compare the recovery trajectories of successful endoscopic CTR (not requiring revision) to successful open CTR.

Open CTR causes more severe postoperative symptoms than endoscopic CTR over the first 1–2 weeks after surgery. Beyond 3 weeks, cohorts of patients receiving open CTR may see greater improvements from baseline than those receiving endoscopic CTR, although the current literature does not demonstrate this with a high degree of certainty. Patients can continue to improve for 24 weeks or longer following either procedure. Future RCTs are required to compare recovery after these interventions, with a focus on both early (first 3 weeks) and late (beyond 24 weeks) outcomes.

## Supplementary Material

zraf085_Supplementary_Data

## Data Availability

Publicly available data sets and analysis scripts supporting the conclusions of this study have been deposited in GitHub and can be accessed via https://github.com/liv-hartrick/Time-to-recovery-following-open-and-endoscopic-carpal-tunnel-decompression-a-systematic-review.

## Supplementary material

*Supplementary*  *material* is available at *BJS Open* online.
