# Supplementary material for: Time to recovery following open and endoscopic carpal tunnel decompression: meta-analysis
Source: BJS Open. 2025 Jul 23;9(4):zraf085. doi: 10.1093/bjsopen/zraf085 (PMC12284922; doi:10.1093/bjsopen/zraf085)
Supplement: zraf085_Supplementary_Data [file zraf085_supplementary_data.docx]

**Recovery following open and endoscopic carpal tunnel release: a systematic review and meta-analysis.**

Ms Olivia J Hartrick, Department of Plastic surgery, Oxford University Hospitals NHS trust; No funding for research or publication.

Ms Rebecca K Turner; UK Centre for Ecology and Hydrology; Durrell Institute of Conservation and Ecology, University of Kent; RKT is funded by the Natural Environment Research Council and the ARIES DTP [Grant Number NE/S007334/1].

Mr Alexander Freethy; Department of Plastic surgery; Royal Devon University Healthcare NHS Foundation Trust; No funding for research or publication.

Mr Chetan Khatri; Trauma and Orthopaedic surgery, University Hospitals Coventry and Warwickshire NHS Trust; Chetan Khatri received a salary as a clinical research fellow as a part of the RACER Knee trials. As a part of the RACER Knee trial, Stryker, a medical device company, fund some treatment costs and imaging costs. This manuscript received no funding from Stryker. The independence of the research is protected by legal agreements and have no bearing on the presented study.

Miss Lauren Chong; School of Medicine and Biomedical Sciences, University of Oxford. No funding for research or publication.

Mr Ryckie G Wade; University of Leeds; Ryckie Wade is funded by the National Institute for Health Research (NIHR CL-2021-02-002).

Mr Justin C R Wormald; NDORMS, University of Oxford; No funding for research or publication.

Mr Akira Wiberg; NDORMS, University of Oxford; No funding for research or publication.

Professor Jeremy N Rodrigues; University of Warwick; No funding for research or publication.

Mr Conrad Harrison; NDORMS, University of Oxford; Email: conrad.harrison@ndorms.ox.ac.uk Address: Department of Plastic surgery, West Wing, John Radcliffe Hospital, Headley Way, Headington, Oxford OX3 9DU. No funding for research or publication.

**Supplementary Materials**

**Supplementary Methods**

| S1: Search Strategy Detail: this appendix includes the terms and Boolean operators used to search databases, the dates searched, and the number of results exported. | Page. 6 |
| --- | --- |
| S2: Risk of Bias Assessment  Detail: this appendix includes the rationale and modified version of the NIH quality assessment tool which was used to assess risk of bias in this study | Page. 12 |
| S3: Calculations for cumulative standardised mean change confidence intervals approximations | Page. 13 |
| **Supplementary Figures** |  |
| S1: A: Standardised mean change for Open CTR BCTQ score at 1 week (I^2^ = 0%). B: funnel plot sensitivity analysis.  S2: A: Standardised mean change for Open CTR BCTQ score at 2 weeks (I^2^ = 91%). B: funnel plot sensitivity analysis.  S3: A: Standardised mean change for Open CTR BCTQ score at 3 weeks (I^2^ = 0%). B: funnel plot sensitivity analysis.  S4: A: Standardised mean change for Open CTR BCTQ score at 4 weeks (I^2^ = 0%). B: funnel plot sensitivity analysis.  S5: A: Standardised mean change for Open CTR BCTQ score at 6 weeks (I^2^ = 90%). B: funnel plot sensitivity analysis.  S6: A: Standardised mean change for Open CTR BCTQ score at 12 weeks (I^2^ = 71%). B: funnel plot sensitivity analysis.  S7: A: Standardised mean change for Open CTR BCTQ score at 24 weeks (I^2^ = 89%). B: funnel plot sensitivity analysis.  S8: A: Standardised mean change for Open CTR BCTQ score at 52 weeks (I^2^ = 95%). B: funnel plot sensitivity analysis.  S9: A: Standardised mean change for Open CTR BCTQ score at 72 weeks (I^2^ = 66%). B: funnel plot sensitivity analysis.  S10: A: Standardised mean change for Open CTR BCTQ score at 104 weeks (I^2^ = 68%). B: funnel plot sensitivity analysis.  S11 A: Standardised mean change for Endoscopic CTR BCTQ score at 1 week (I^2^ = 95%). B: funnel plot sensitivity analysis.  S12 A: Standardised mean change for Endoscopic CTR BCTQ score at 2 weeks (I^2^ = 94%). B: funnel plot sensitivity analysis.  S13 A: Standardised mean change for Endoscopic CTR BCTQ score at 3 weeks (I^2^ = 7%). B: funnel plot sensitivity analysis.  S14: A: Standardised mean change for Endoscopic CTR BCTQ score at 4 weeks (I^2^ = 14%). B: funnel plot sensitivity analysis.  S15: A: Standardised mean change for Endoscopic CTR BCTQ score at 6 weeks (I^2^ = 0%). B: funnel plot sensitivity analysis.  S16: A: Standardised mean change for Endoscopic CTR BCTQ score at 12 weeks (I^2^ = 0%). B: funnel plot sensitivity analysis.  S17: A: Standardised mean change for Endoscopic CTR BCTQ score at 24 weeks (I^2^ = 31%). B: funnel plot sensitivity analysis.  S18: A: Standardised mean change for Endoscopic CTR BCTQ score at 52 weeks (I^2^ = 95%). B: funnel plot sensitivity analysis.  S19: A: Standardised mean change for Endoscopic CTR BCTQ score at 72 weeks (I^2^ = 0%). B: funnel plot sensitivity analysis.  S20: A: Standardised mean change for Endoscopic CTR BCTQ score at 104 weeks (I^2^ = 0%). B: funnel plot sensitivity analysis. | Page. 14  Page. 15  Page. 16  Page. 17  Page. 18  Page. 19  Page. 20  Page. 21  Page. 22  Page. 23  Page. 24  Page. 25  Page. 26  Page. 27  Page. 28  Page. 29  Page. 30  Page. 31  Page. 32  Page. 33 |
| S21: Change in qDASH score for open and endoscopic carpal tunnel release over time. Each line represents a study arm. A decrease in score over time indicates recovery to a plateau. | Page. 34 |
| **Supplementary Table** |  |
| S1: Summary of the characteristics of included studies. | Page. 36 |
| S2: Risk of bias assessment results of all included studies | Page. 40 |
| S3: Table S5: Cumulative Standardised mean change (SMC) in Boston Carpal Tunnel Questionnaire scores from baseline for open and endoscopic carpal tunnel release (CTR) at 1, 2, 3, 4, 6, 12, 24, 52, 72 AND 104-week timepoints. | Page. 42 |

**Supplementary Methods**

**S1: Search strategy**

PICO framework

| Table S2 shows the framework of search terms which were used to search databases^41^. | | | |
| --- | --- | --- | --- |
| **Population** | **Intervention** | **Comparator** | **Outcome** |
| “Carpal tunnel syndrome” | "surgery"  "operation"  "release"  "decompression"  **MeSH/keywords:**  “microvascular decompression surgery”  “surgery, plastic” | **-** | "recover*"  "outcome"  "recovery outcome*"  **MeSH/Keyword:**  "Recovery of function" |

**Initial search**

Database: Pubmed

Date searched: 04.07.2023

Results: 114

Search terms:

("carpal tunnel syndrome"[MeSH Terms] OR "carpal tunnel syndrome"[Title/Abstract])

AND

("microvascular decompression surgery"[MeSH Terms] OR "surgery, plastic"[MeSH Terms] OR "surgery"[Title/Abstract] OR "operation"[Title/Abstract] OR "release"[Title/Abstract] OR "decompression"[Title/Abstract])

AND

(("recover*"[Title/Abstract] OR "outcome"[Title/Abstract] OR "recovery outcome*"[Title/Abstract]) AND "recovery of function"[MeSH Terms])

Database: Medline via OVID

Date searched: 04.07.2023

Results: 1362

Search terms:

carpal tunnel syndrome.ti. or carpal tunnel syndrome.ab. or carpal tunnel syndrome.sh. or carpal tunnel syndrome.kw.

AND

(surgery or operation or release or decompression).ti. or (surgery or operation or release or decompression).ab. or ("microvascular decompression surgery" or "surgery, plastic").sh. or ("microvascular decompression surgery" or "surgery, plastic").kw.

AND

(recover* or outcome* or "recovery outcome*").ti. or (recover* or outcome* or "recovery outcome*").ab. or "recovery of function".sh. or "recovery of function".kw.

Database: EMBASE

Date searched: 04.07.2023

Results: 1770

Search terms:

3

(surgery or operation or release or decompression).ab. or (surgery or operation or release or decompression).ti. or ("microvascular decompression surgery" or "surgery, plastic").sh. or ("microvascular decompression surgery" or "surgery, plastic").kw.

3040822

4

(recover* or outcome* or "recovery outcome*").ab. or (recover* or outcome* or "recovery outcome*").ti. or "recovery of function".sh. or "recovery of function".kw.

4353143

5

2 and 3 and 4

4

(recover* or outcome* or "recovery outcome*").ab. or (recover* or outcome* or "recovery outcome*").ti. or "recovery of function".sh. or "recovery of function".kw.

5

2 and 3 and 4

Database: Cochrane library

Date searched: 04.07.2023

Results: 15

Search terms:

"carpal tunnel syndrome" in Title Abstract Keyword AND "surgery" OR "operation" OR "release" OR "decompression" in Title Abstract Keyword AND "recover*" OR "outcome*" OR "recovery outcome*" in Title Abstract Keyword - (Word variations have been searched)

**Second search**

Database: Pubmed

Date searched 21.08.2024

Results: 3

Search terms:

| Search number | Query | Sort By | Filters | Search Details | Results |
| --- | --- | --- | --- | --- | --- |
| 5 | #1 AND #2 AND #3 | | from 2023/7/5 - 2024/8/22 | (("carpal tunnel syndrome"[Title/Abstract] OR "carpal tunnel syndrome"[MeSH Terms]) AND ("microvascular decompression surgery"[MeSH Terms] OR "surgery, plastic"[MeSH Terms] OR "surgery"[Title/Abstract] OR "operation"[Title/Abstract] OR "release"[Title/Abstract] OR "decompression"[Title/Abstract]) AND (("recover*"[Title/Abstract] OR "outcome"[Title/Abstract] OR "recovery outcome*"[Title/Abstract]) AND "recovery of function"[MeSH Terms])) AND (2023/7/5:2024/8/22[pdat]) | 3 |
| 4 | #1 AND #2 AND #3 | |  | ("carpal tunnel syndrome"[Title/Abstract] OR "carpal tunnel syndrome"[MeSH Terms]) AND ("microvascular decompression surgery"[MeSH Terms] OR "surgery, plastic"[MeSH Terms] OR "surgery"[Title/Abstract] OR "operation"[Title/Abstract] OR "release"[Title/Abstract] OR "decompression"[Title/Abstract]) AND (("recover*"[Title/Abstract] OR "outcome"[Title/Abstract] OR "recovery outcome*"[Title/Abstract]) AND "recovery of function"[MeSH Terms]) | 117 |
| 3 | (("recover*"[Title/Abstract] OR "outcome"[Title/Abstract] OR "recovery outcome*"[Title/Abstract]) AND "recovery of function"[MeSH Terms]) | | | ("recover*"[Title/Abstract] OR "outcome"[Title/Abstract] OR "recovery outcome*"[Title/Abstract]) AND "recovery of function"[MeSH Terms] | 38,241 |
| 2 | ("microvascular decompression surgery"[MeSH Terms] OR "surgery, plastic"[MeSH Terms] OR "surgery"[Title/Abstract] OR "operation"[Title/Abstract] OR "release"[Title/Abstract] OR "decompression"[Title/Abstract]) | | | "microvascular decompression surgery"[MeSH Terms] OR "surgery, plastic"[MeSH Terms] OR "surgery"[Title/Abstract] OR "operation"[Title/Abstract] OR "release"[Title/Abstract] OR "decompression"[Title/Abstract] | 2,596,330 |
| 1 | (carpal tunnel syndrome[Title/Abstract]) OR (carpal tunnel syndrome[MeSH Terms]) | | | "carpal tunnel syndrome"[Title/Abstract] OR "carpal tunnel syndrome"[MeSH Terms] | 13,085 |

Database: Ovid MEDLINE(R) ALL <1946 to August 21, 2024>

Date searched 21.08.2024

Results: 213

Search terms:

1 carpal tunnel syndrome.ti. or carpal tunnel syndrome.ab. or carpal tunnel syndrome.hw. or carpal tunnel syndrome.kw. 13084

2 (surgery or operation or release or decompression).ti. or (surgery or operation or release or decompression).ab. or ("microvascular decompression surgery" or "surgery, plastic").hw. or ("microvascular decompression surgery" or "surgery, plastic").kw. 2476263

3 (recover* or outcome* or "recovery outcome*").ti. or (recover* or outcome* or "recovery outcome*").ab. or "recovery of function".hw. or "recovery of function".kw. 3328253

4 1 and 2 and 3 1524

5 limit 4 to yr="2023 - 2024" 213

Database: Embase <1974 to 2024 August 21>

Date searched: 21.08.2024

Results: 224

Search terms:

1 carpal tunnel syndrome.ti. or carpal tunnel syndrome.ab. or carpal tunnel syndrome.sh. or carpal tunnel syndrome.kw. 19908

2 (surgery or operation or release or decompression).ti. or (surgery or operation or release or decompression).ab. or ("microvascular decompression surgery" or "surgery, plastic").sh. or ("microvascular decompression surgery" or "surgery, plastic").kw. 3300940

3 (recover* or outcome* or "recovery outcome*").ti. or (recover* or outcome* or "recovery outcome*").ab. or "recovery of function".sh. or "recovery of function".kw. 4732266

4 1 and 2 and 3 1896

5 limit 4 to yr="2023 - 2024" 224

Database: Cochrane library

Date searched: 21.08.2024

Results: 582

Search terms:

"carpal tunnel syndrome" in Title Abstract Keyword AND "surgery" OR "operation" OR "release" OR "decompression" in Title Abstract Keyword AND "recover*" OR "outcome*" OR "recovery outcome*" in Title Abstract Keyword - (Word variations have been searched)

**S2: Risk of Bias Assessment**

The standardised risk of bias (ROB) tools which assess ROB in systematic reviews for comparative studies, like ROB2^82^ and ROBINS-1^83^, did not align with our analysis of standardised mean change (SMC). Instead, we modified the National Institute for Health (NIH) quality assessment tool for “Before-After (Pre-Post) Studies With No Control Group”^17^. This tool focused on the specific bias which may have influenced SMC scores at each timepoint in our meta-analysis.

The following questions were used to assess ROB for the included studies:

Q1: Was the study question/objective clearly stated?

Q2: Were eligibility/selection criteria for the study population prespecified and clearly described?

Q3: Were the participants in the study representative of those who would be eligible for the test/service/intervention in the general or clinical population of interest?

Q4: Were all eligible participants that met the prespecified entry criteria enrolled?

Q5: Was the sample size sufficiently large to provide confidence in the findings?

Q6: Was the intervention clearly described and delivered consistently across the study population?

Q7: Were the outcome measures prespecified, clearly defined, valid, reliable, and assessed consistently across all study participants?

Q8: Were the people assessing the outcomes blinded to the participants' interventions?

Q9: Was the loss to follow-up after baseline 20% or less and it so, where those lost to follow-up accounted for in the analysis?

Studies were assessed to have low bias if all questions were answered yes, moderate if one question was answered no and high if two or more questions were answered no.

**S3: Calculations for cumulative standardised mean change confidence intervals approximations**

To account for propagating uncertainty in the trajectory of the cumulative standardised mean change (SMC) estimates shown in Figure 3, we applied a Wald-based correction to the 95% confidence intervals ($CI$), which assumes a normal distribution. We approximated the variance ($SE$) at each SMC time point using the squared width of the raw SMC 95% CI, divided by two:

$$SE= \left( \frac{Upper CI-Lower CI}{2 \times1.96} \right)^{2}$$

We summed the variances across time points to calculate the cumulative variance and derived a cumulative standard error (${SE}_{cumulative}$) as the root of the sum of $SE$ . The 95% confidence intervals (${CI}_{cumulative}$) were then approximated for each cumulative SMC estimate (${SMC}_{cumulative}$) as:

$${CI}_{cumulative}={SMC}_{cumulative} \pm1.96 \times{SE}_{cumulative}$$

Thereby allowing the confidence intervals to widen appropriately with uncertainty across time points.

**Supplementary Figures**

**S1: A: Standardised mean change for Open CTR BCTQ score at 1 week (I^2^ = 0%). B: funnel plot sensitivity analysis.**

**
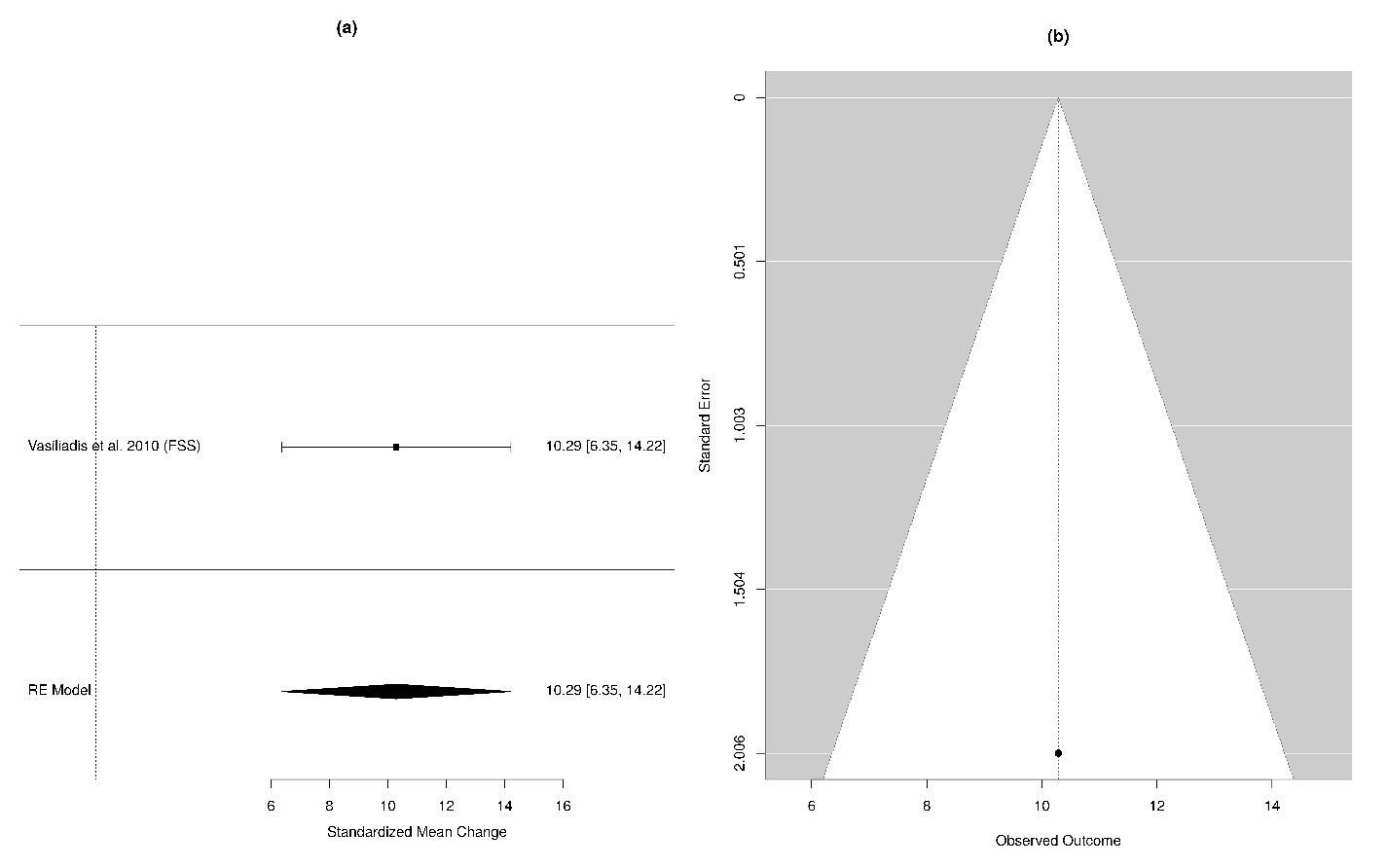
**

Figure S1: A: Standardised mean change for Open CTR BCTQ score at 1 week (I^2^ = 0%). B: funnel plot sensitivity analysis. RE: random effects; FSS: Functional Severity Score; SSS: Symptom Severity Score.

**S2: A: Standardised mean change for Open CTR BCTQ score at 2 weeks (I^2^ = 91%). B: funnel plot sensitivity analysis.**


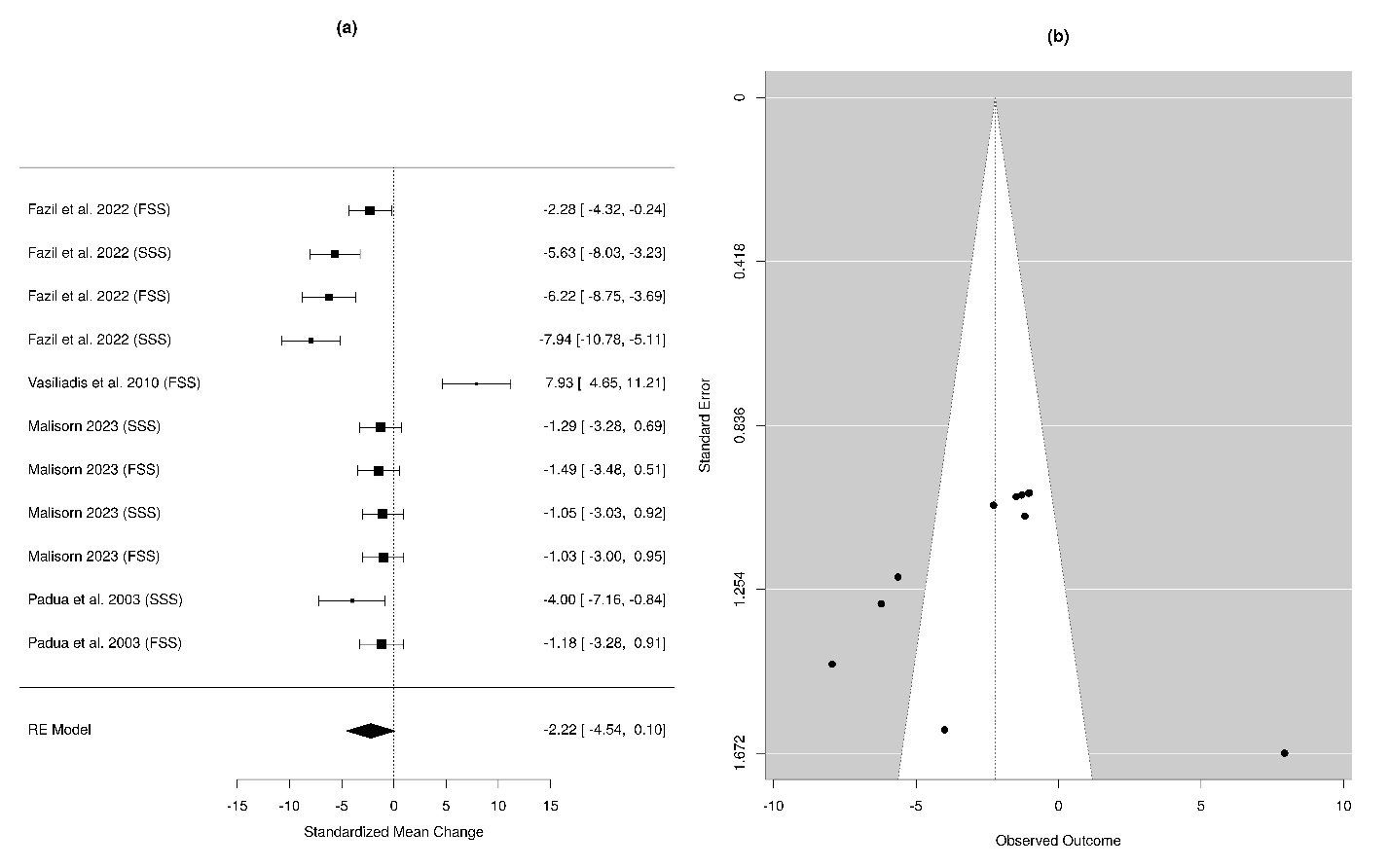


Figure S2: A: Standardised mean change for Open CTR BCTQ score at 2 weeks (I^2^ = 91%). B: funnel plot sensitivity analysis. RE: random effects; FSS: Functional Severity Score; SSS: Symptom Severity Score.

**S3: A: Standardised mean change for Open CTR BCTQ score at 3 weeks (I^2^ = 0%). B: funnel plot sensitivity analysis.**


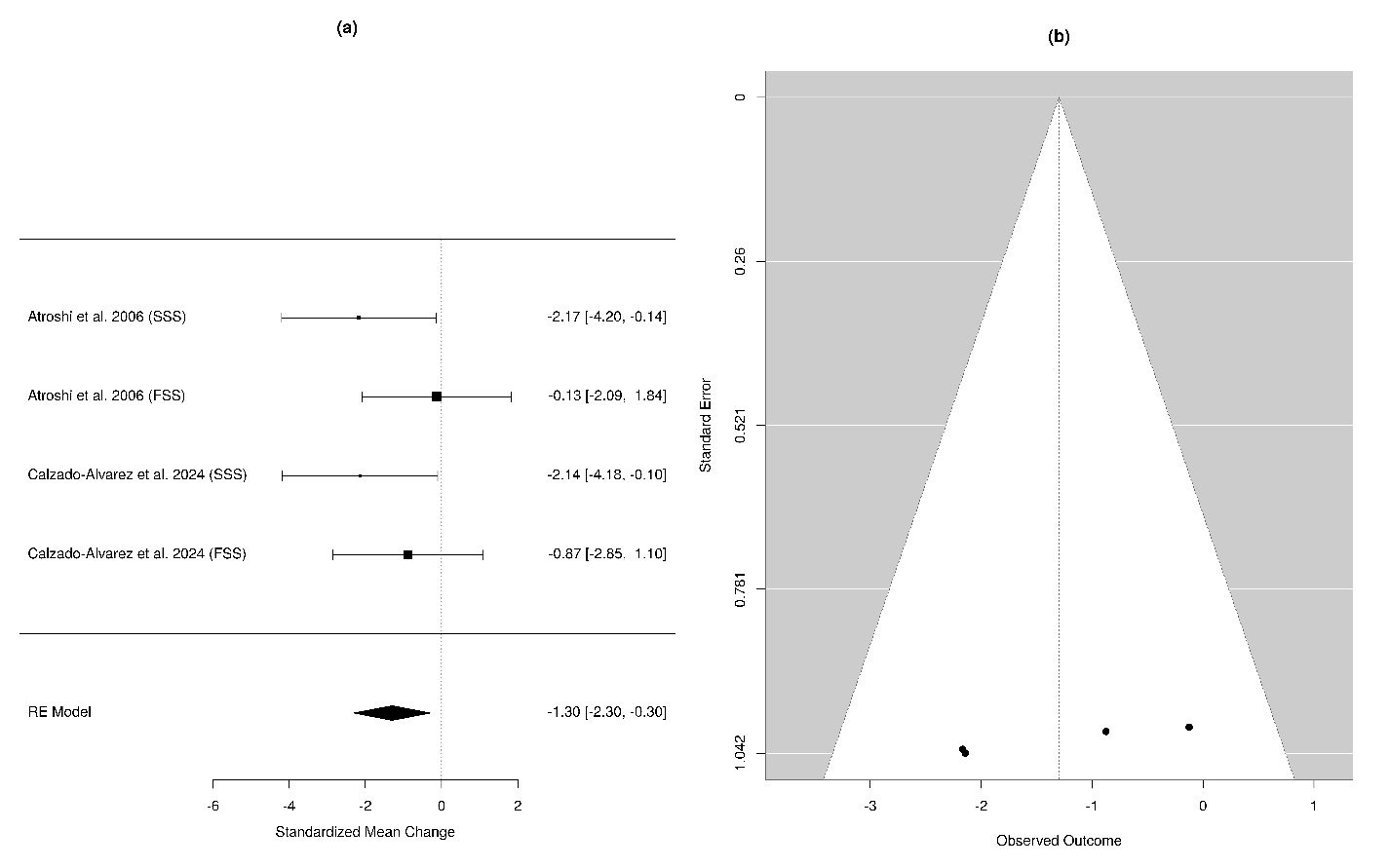


Figure S3: A: Standardised mean change for Open CTR BCTQ score at 3 weeks (I^2^ = 0%). B: funnel plot sensitivity analysis. RE: random effects; FSS: Functional Severity Score; SSS: Symptom Severity Score.

Symptom Severity Score.

**S4: A: Standardised mean change for Open CTR BCTQ score at 4 weeks (I^2^ = 0%). B: funnel plot sensitivity analysis.**


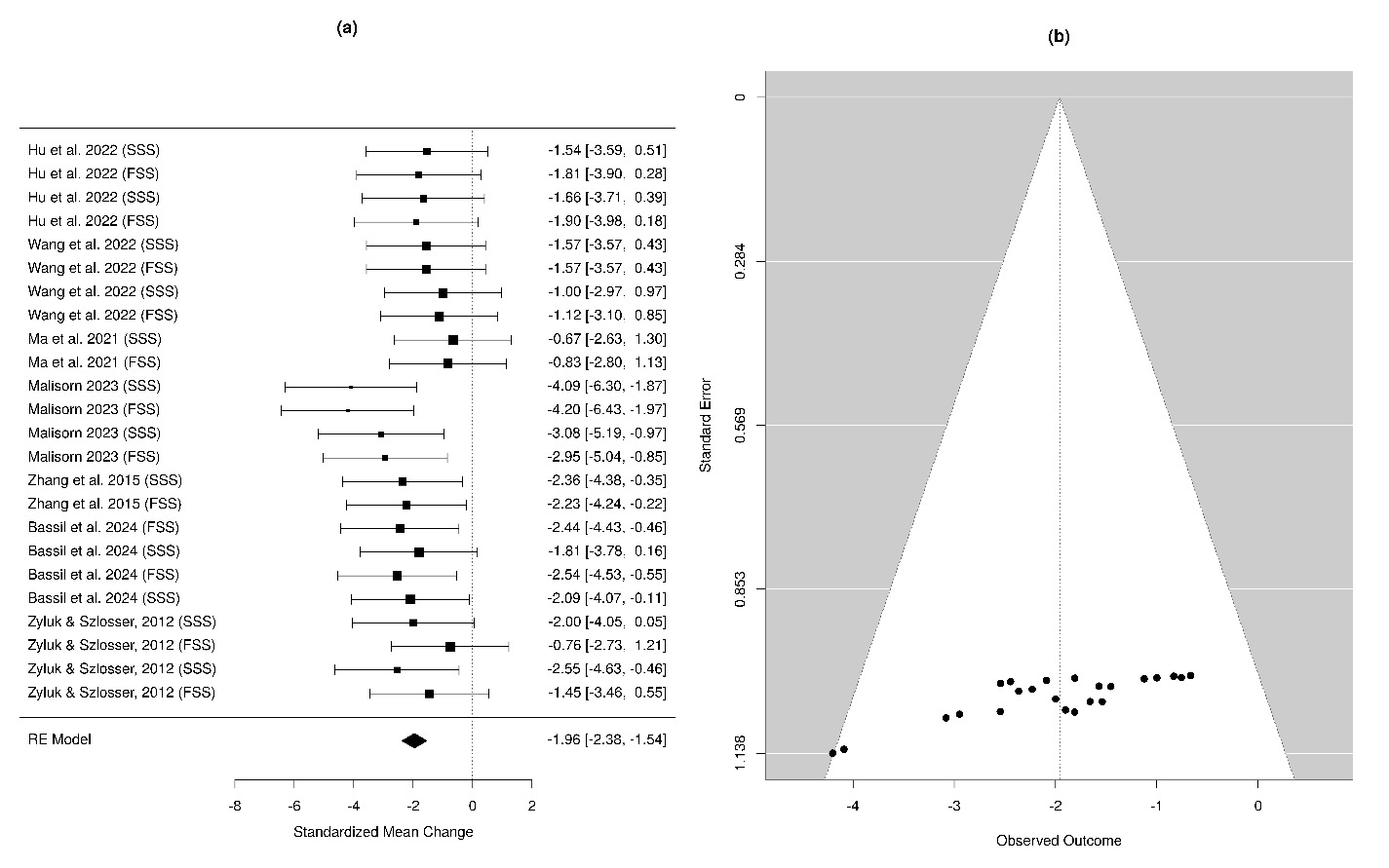


Figure S4: A: Standardised mean change for Open CTR BCTQ score at 4 weeks (I^2^ = 0%). B: funnel plot sensitivity analysis. RE: random effects; FSS: Functional Severity Score; SSS: Symptom Severity Score.

**S5: A: Standardised mean change for Open CTR BCTQ score at 6 weeks (I^2^ = 90%). B: funnel plot sensitivity analysis.**


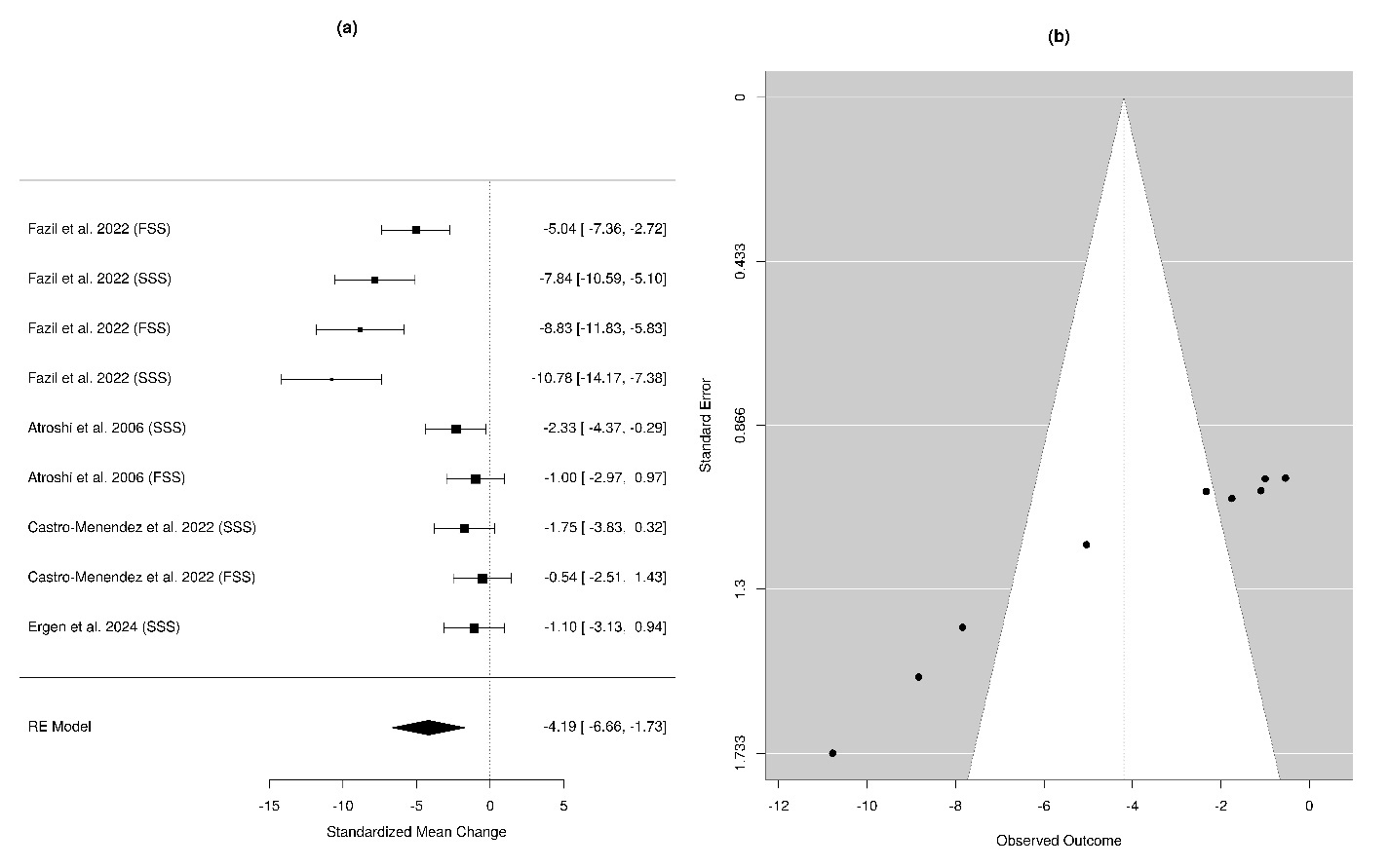


Figure S5: A: Standardised mean change for Open CTR BCTQ score at 6 weeks (I^2^ = 90%). B: funnel plot sensitivity analysis. RE: random effects; FSS: Functional Severity Score; SSS: Symptom Severity Score.

**S6: A: Standardised mean change for Open CTR BCTQ score at 12 weeks (I^2^ = 71%). B: funnel plot sensitivity analysis.**


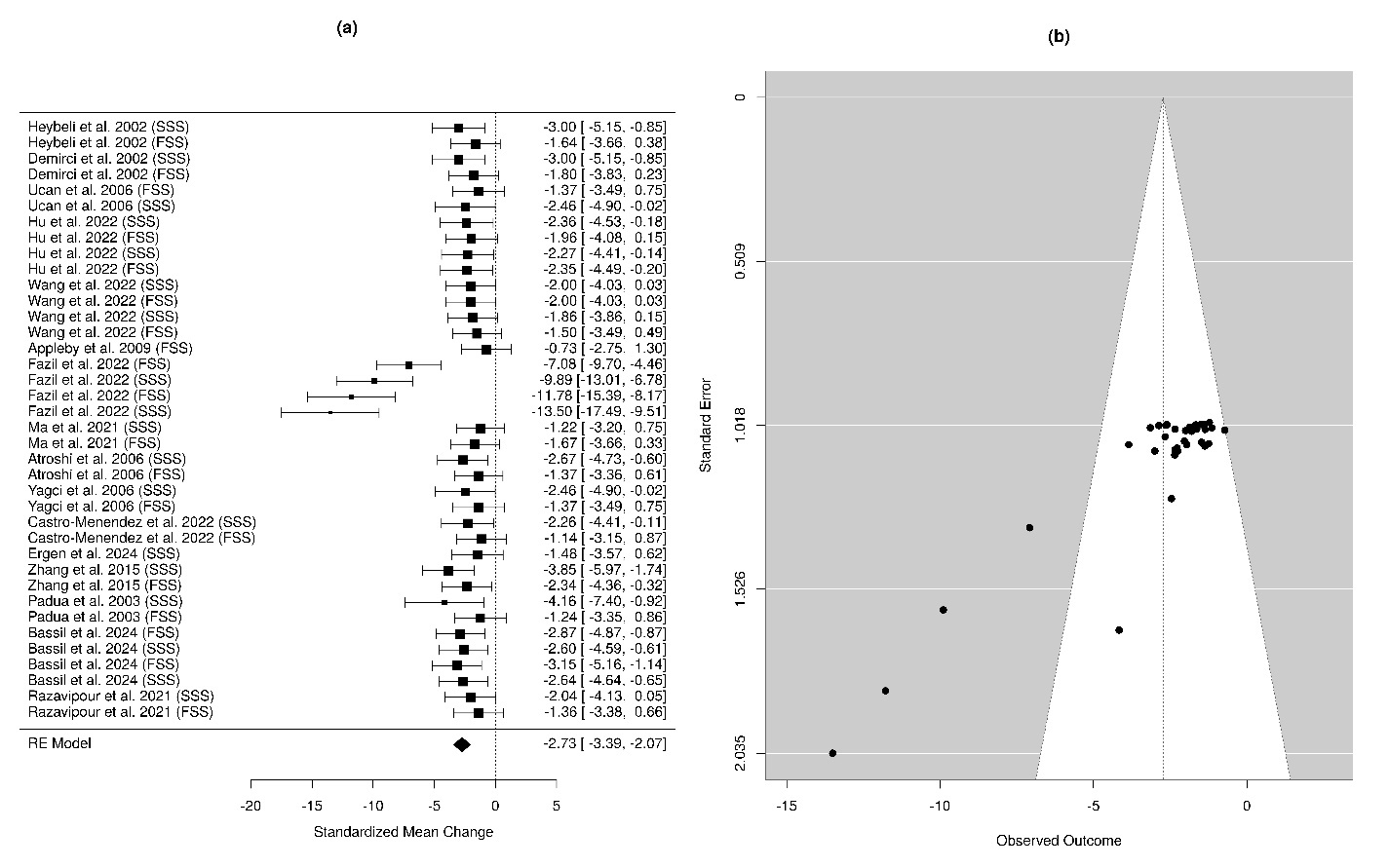


Figure S6: A: Standardised mean change for Open CTR BCTQ score at 12 weeks (I^2^ = 71%). B: funnel plot sensitivity analysis. RE: random effects; FSS: Functional Severity Score; SSS: Symptom Severity Score.

**S7: A: Standardised mean change for Open CTR BCTQ score at 24 weeks (I^2^ = 89%). B: funnel plot sensitivity analysis.**


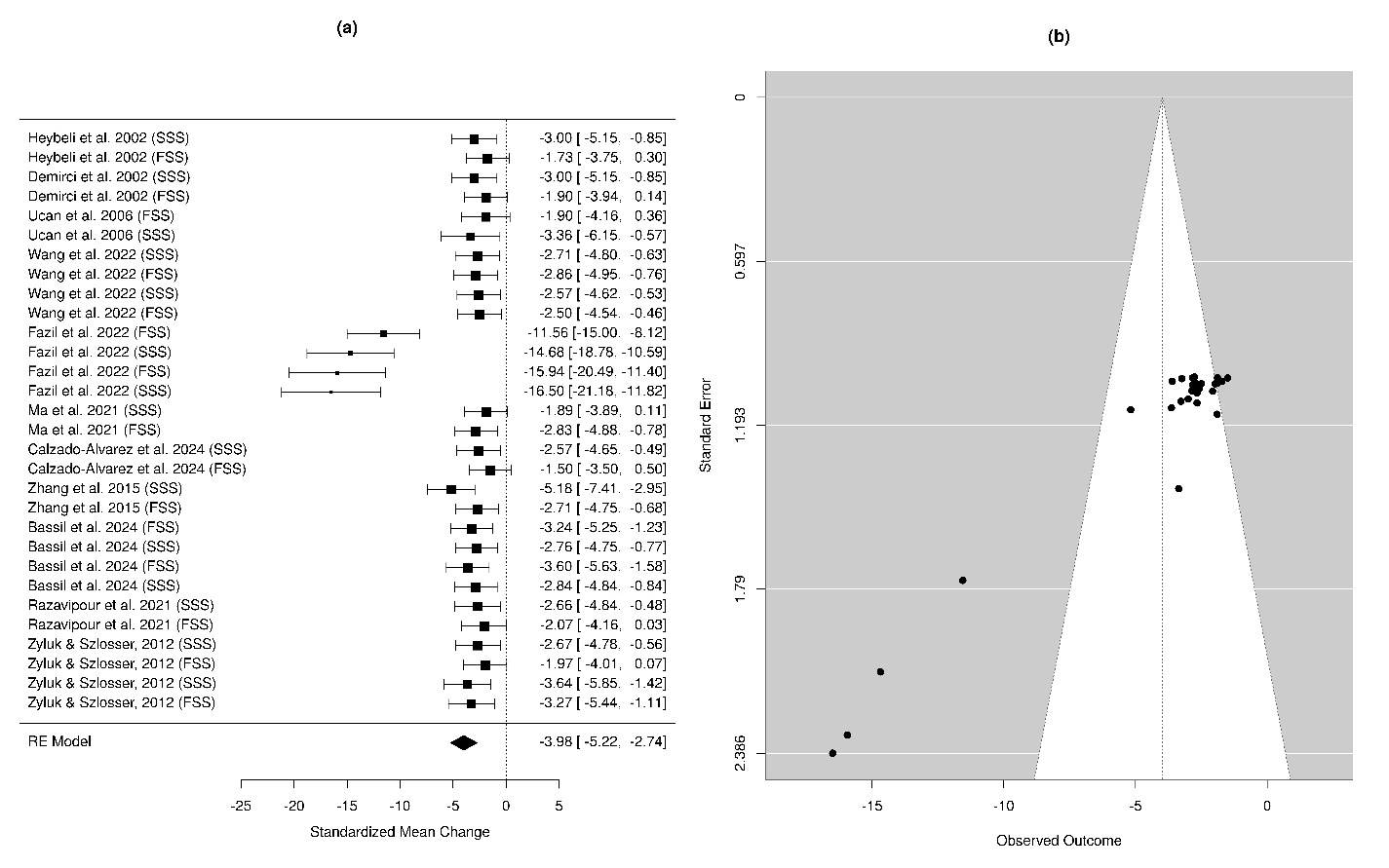


Figure S7: A: Standardised mean change for Open CTR BCTQ score at 24 weeks (I^2^ = 89%). B: funnel plot sensitivity analysis. RE: random effects; FSS: Functional Severity Score; SSS: Symptom Severity Score.

**S8: A: Standardised mean change for Open CTR BCTQ score at 52 weeks (I^2^ = 95%). B: funnel plot sensitivity analysis.**

**
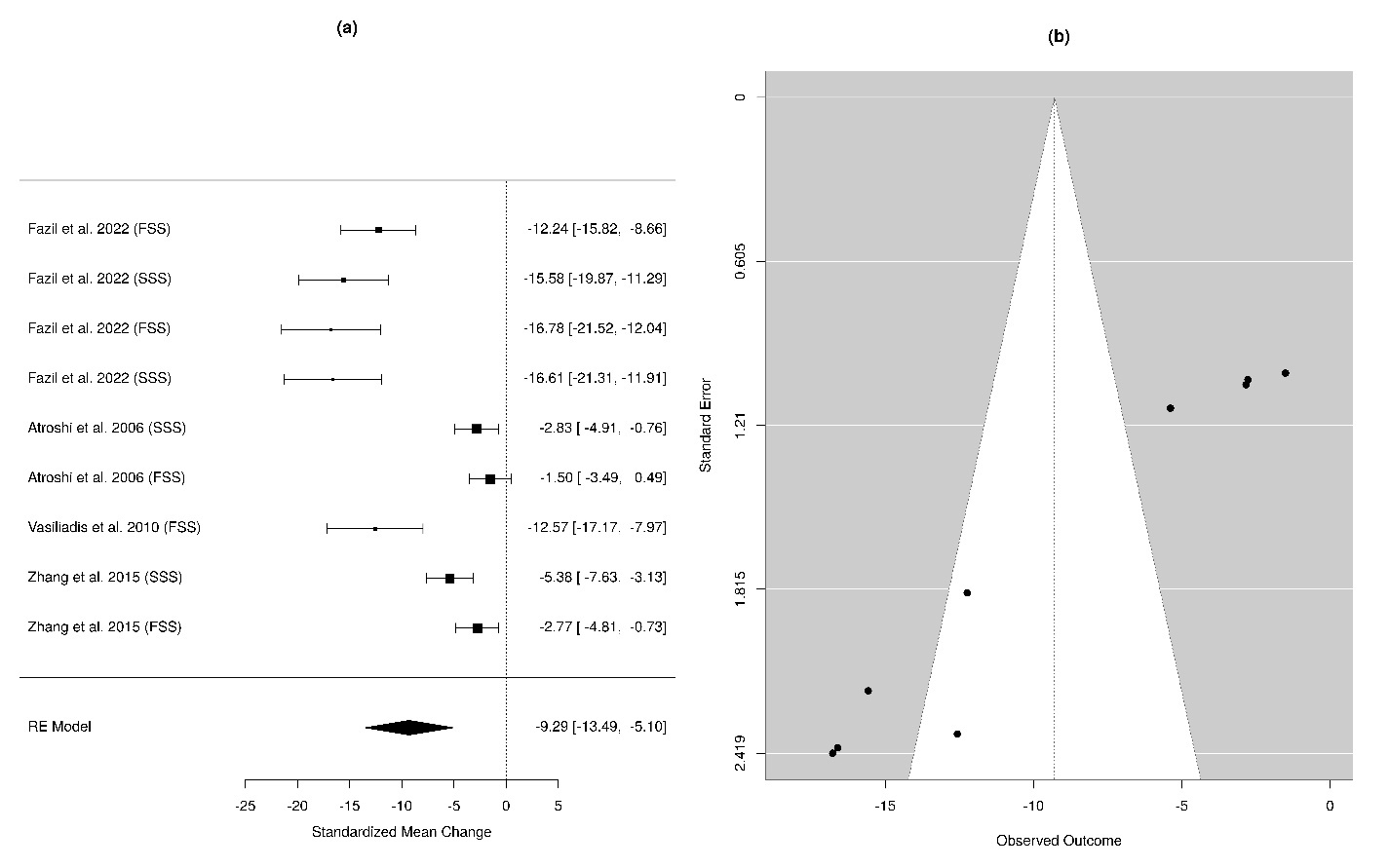
**

Figure S8: A: Standardised mean change for Open CTR BCTQ score at 52 weeks (I^2^ = 95%). B: funnel plot sensitivity analysis. RE: random effects; FSS: Functional Severity Score; SSS: Symptom Severity Score.

**S9: A: Standardised mean change for Open CTR BCTQ score at 72 weeks (I^2^ = 66%). B: funnel plot sensitivity analysis.**

**
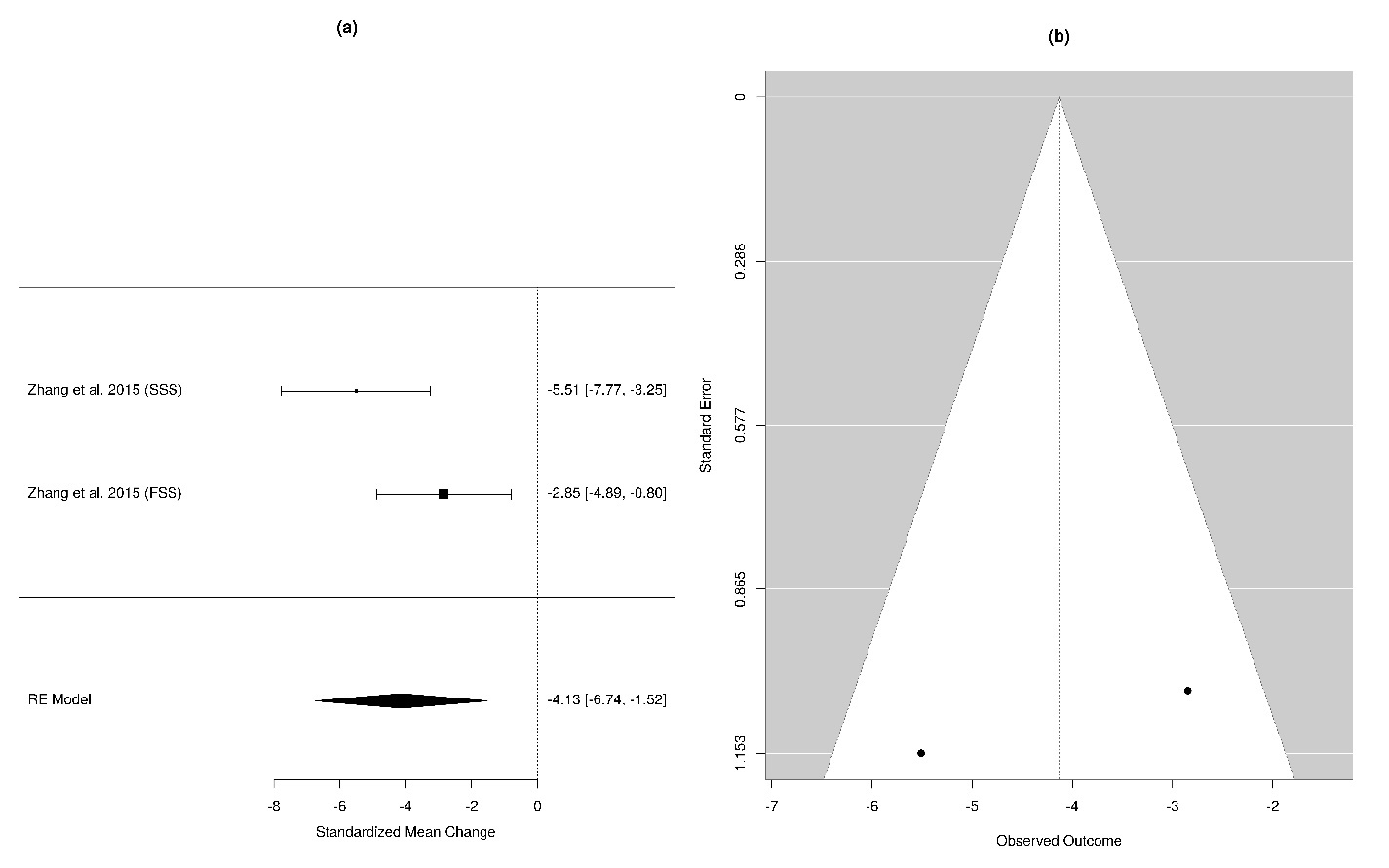
**

Figure S9: A: Standardised mean change for Open CTR BCTQ score at 72 weeks (I^2^ = 66%). B: funnel plot sensitivity analysis. RE: random effects; FSS: Functional Severity Score; SSS: Symptom Severity Score.

**S10: A: Standardised mean change for Open CTR BCTQ score at 104 weeks (I^2^ = 68%). B: funnel plot sensitivity analysis.**

**
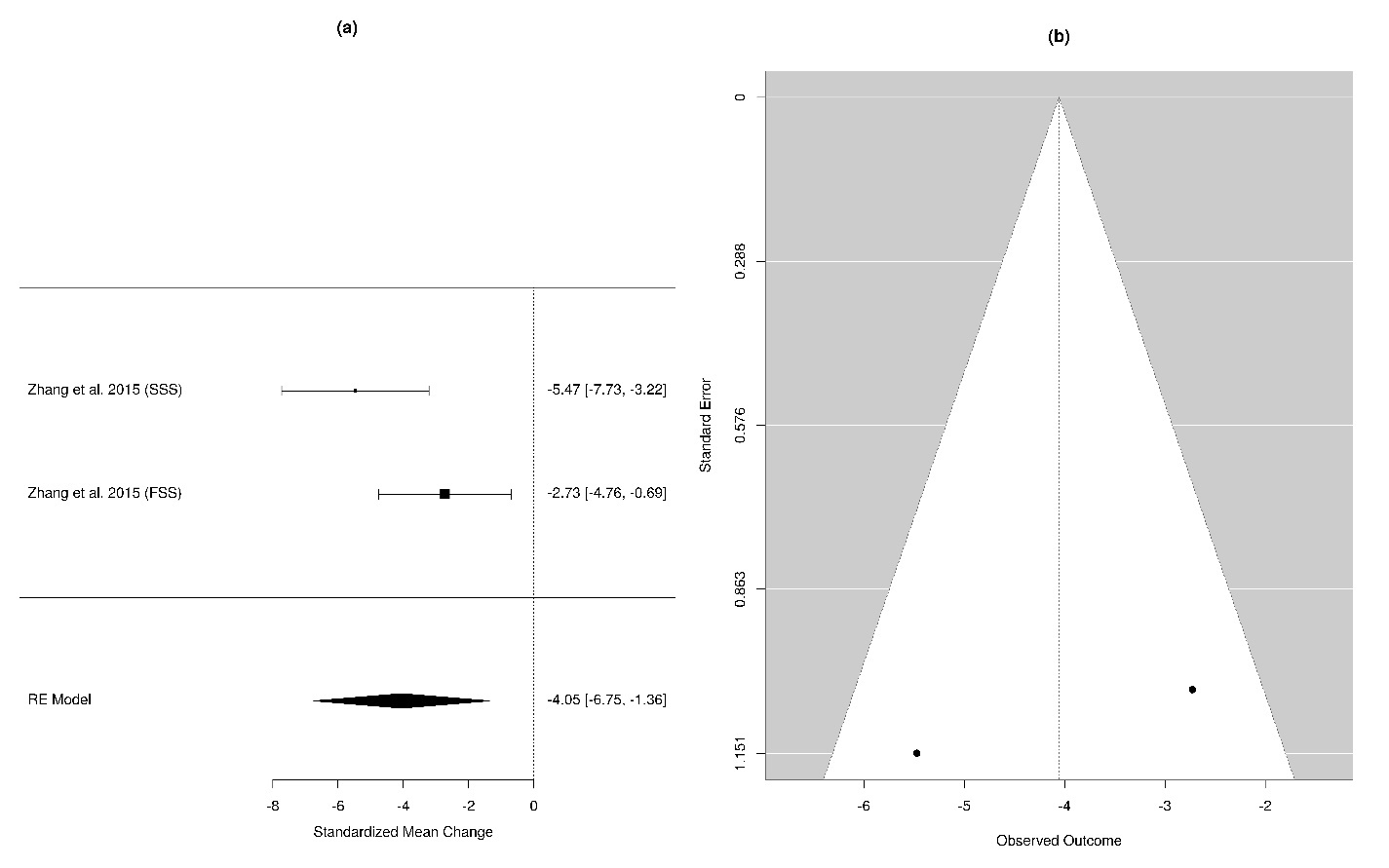
**

Figure S10: A: Standardised mean change for Open CTR BCTQ score at 104 weeks (I^2^ = 68%). B: funnel plot sensitivity analysis. RE: random effects; FSS: Functional Severity Score; SSS: Symptom Severity Score.

**S11 A: Standardised mean change for Endoscopic CTR BCTQ score at 1 week (I^2^ = 95%). B: funnel plot sensitivity analysis.**

**
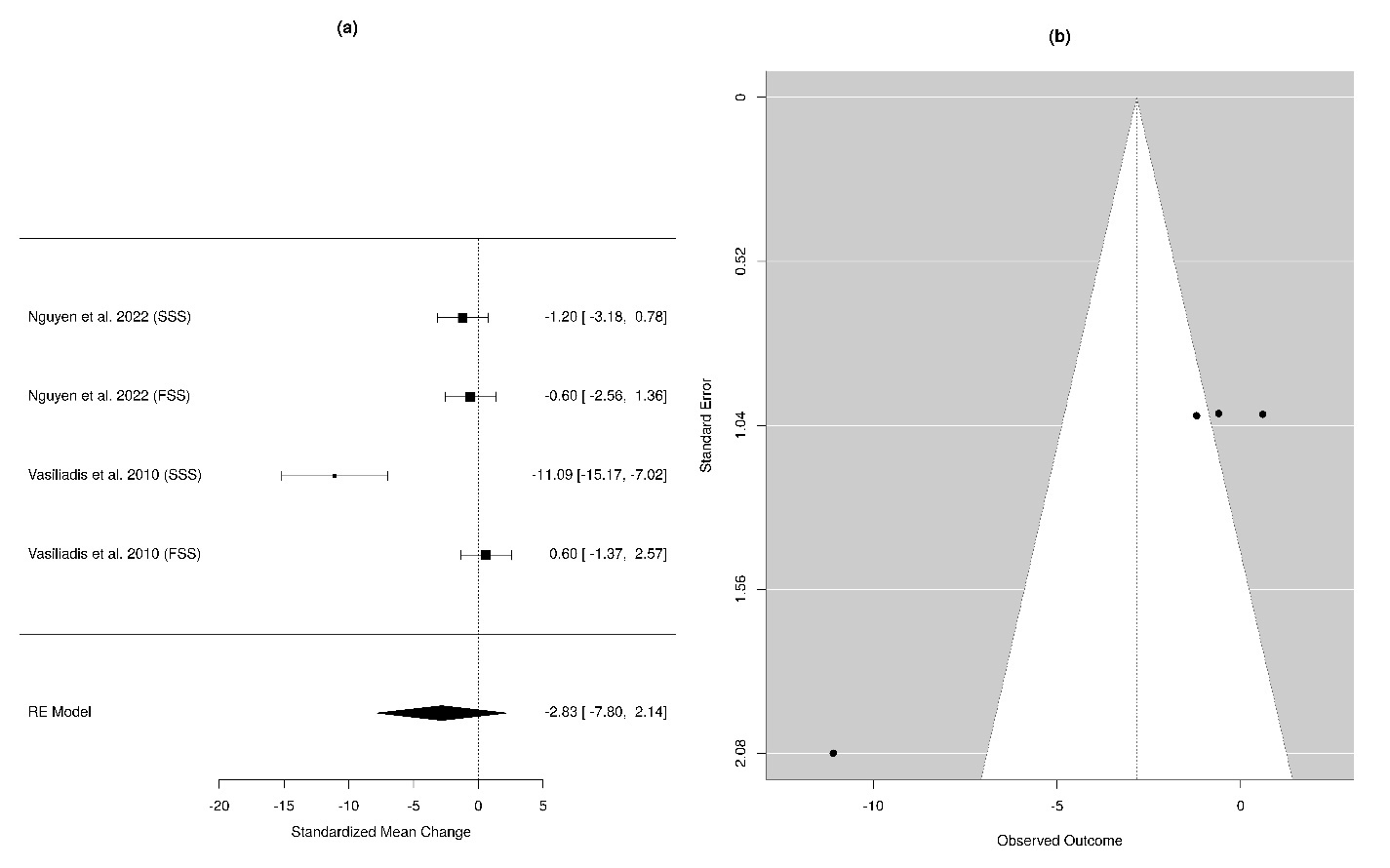
**

Figure S11 A: Standardised mean change for Endoscopic CTR BCTQ score at 1 week (I^2^ = 95%). B: funnel plot sensitivity analysis. RE: random effects; FSS: Functional Severity Score; SSS: Symptom Severity Score.

**S12 A: Standardised mean change for Endoscopic CTR BCTQ score at 2 weeks (I^2^ = 94%). B: funnel plot sensitivity analysis.**

**
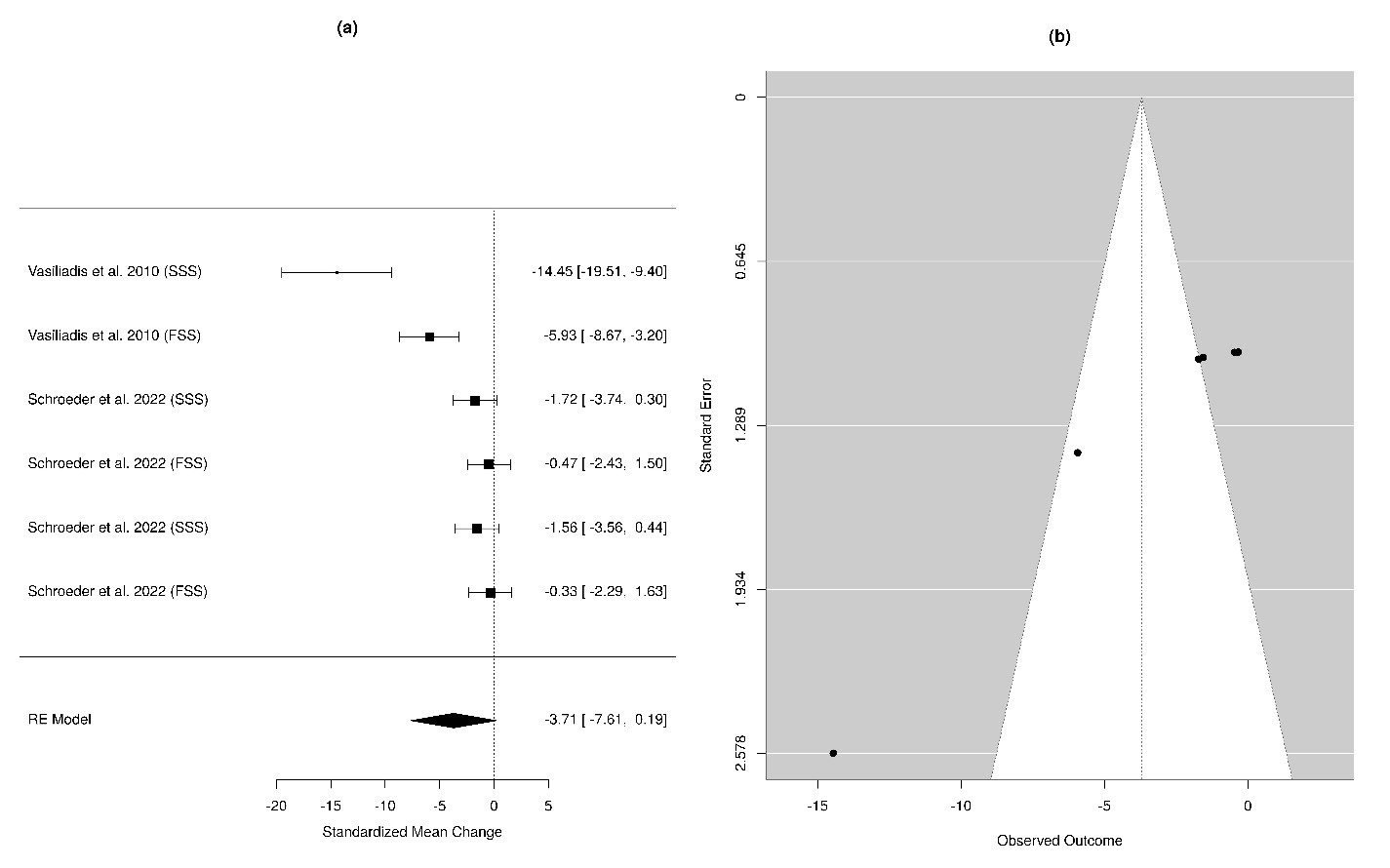
**

Figure S12 A: Standardised mean change for Endoscopic CTR BCTQ score at 2 weeks (I^2^ = 94%). B: funnel plot sensitivity analysis. RE: random effects; FSS: Functional Severity Score; SSS: Symptom Severity Score.

**S13 A: Standardised mean change for Endoscopic CTR BCTQ score at 3 weeks (I^2^ = 7%). B: funnel plot sensitivity analysis.**

**
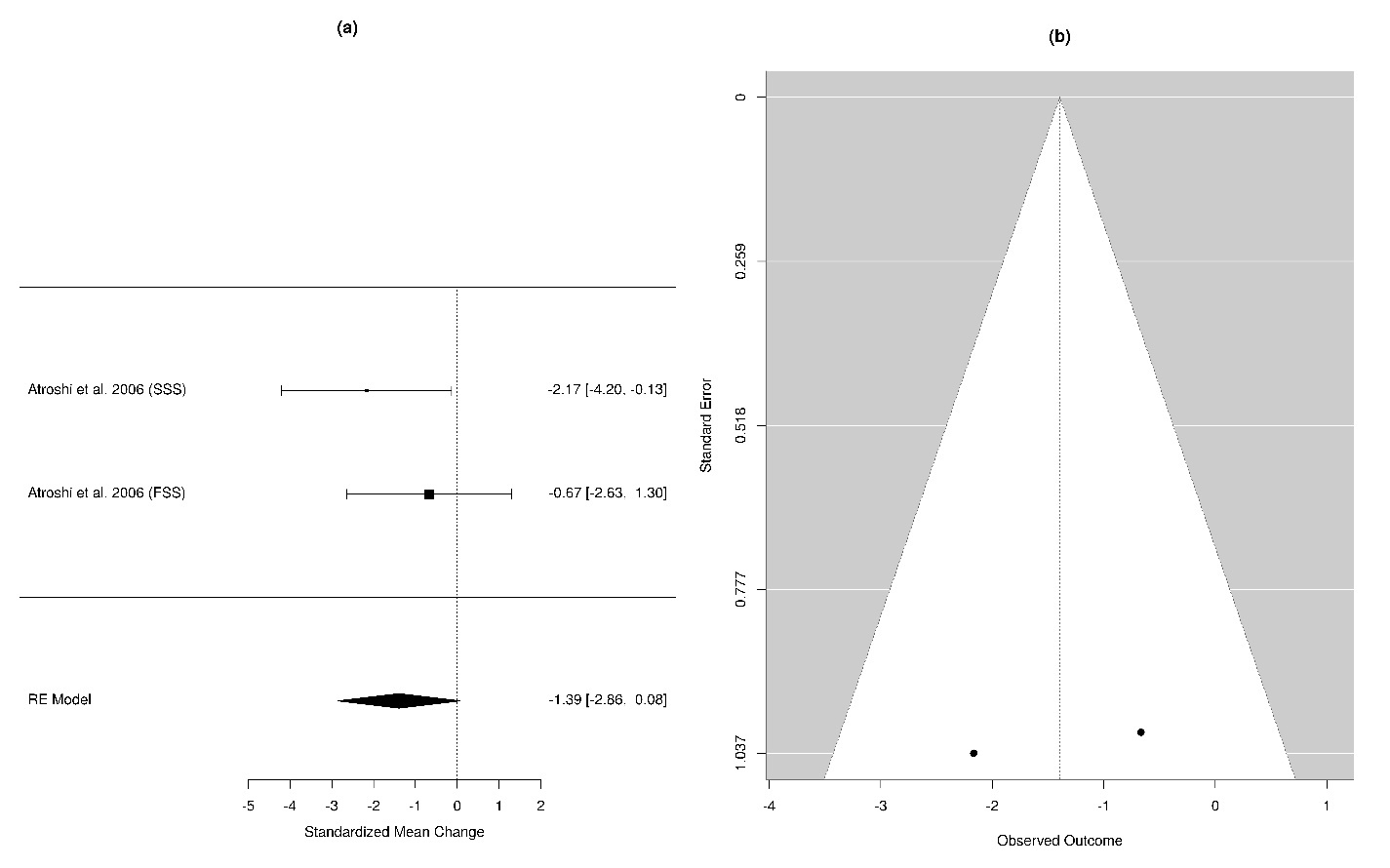
**

Figure S13 A: Standardised mean change for Endoscopic CTR BCTQ score at 3 weeks (I^2^ = 7%). B: funnel plot sensitivity analysis. RE: random effects; FSS: Functional Severity Score; SSS: Symptom Severity Score.

**S14: A: Standardised mean change for Endoscopic CTR BCTQ score at 4 weeks (I^2^ = 14%). B: funnel plot sensitivity analysis.**

**
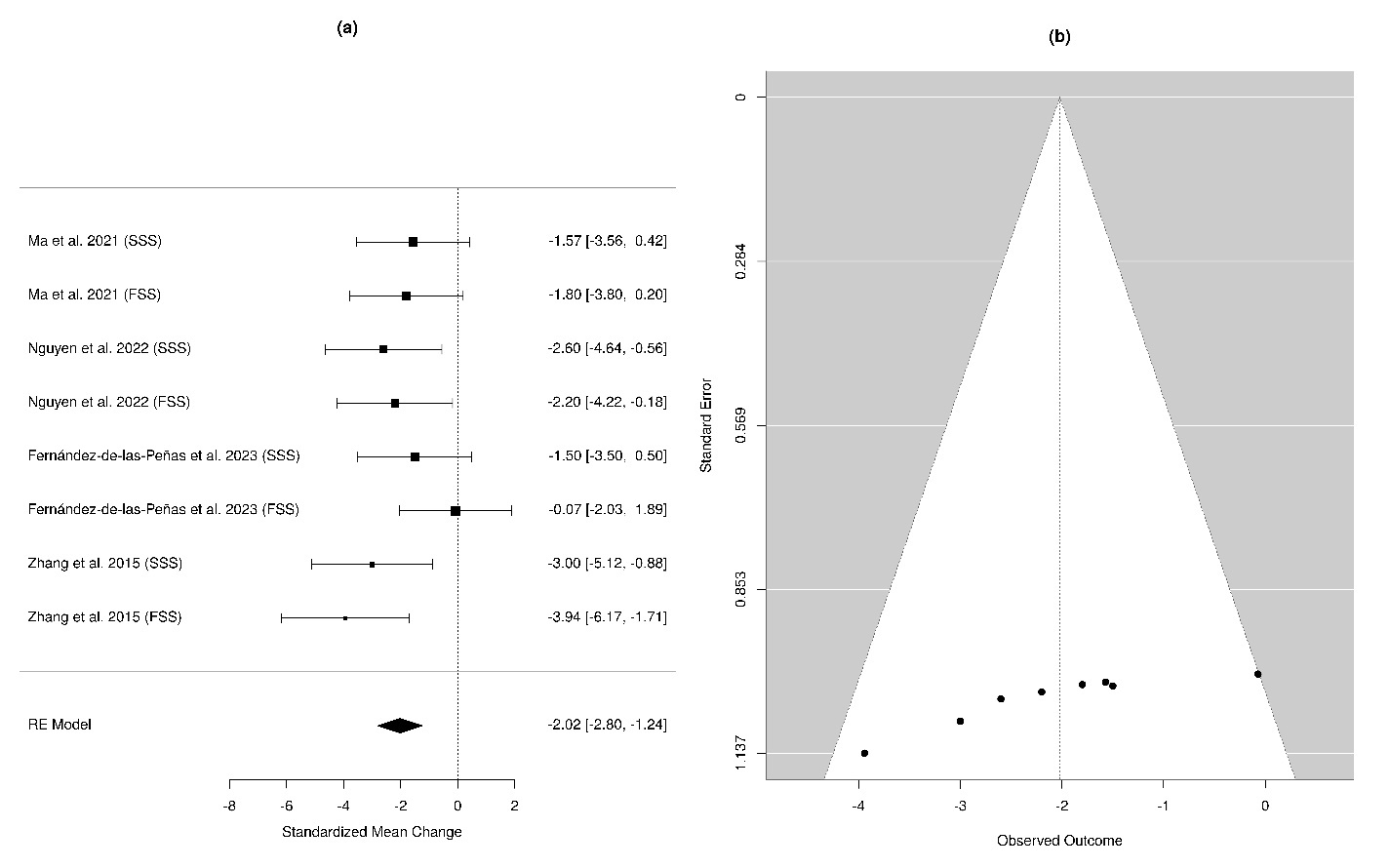
**

Figure S14: A: Standardised mean change for Endoscopic CTR BCTQ score at 4 weeks (I^2^ = 14%). B: funnel plot sensitivity analysis. RE: random effects; FSS: Functional Severity Score; SSS: Symptom Severity Score.

**S15: A: Standardised mean change for Endoscopic CTR BCTQ score at 6 weeks (I^2^ = 0%). B: funnel plot sensitivity analysis.**

**
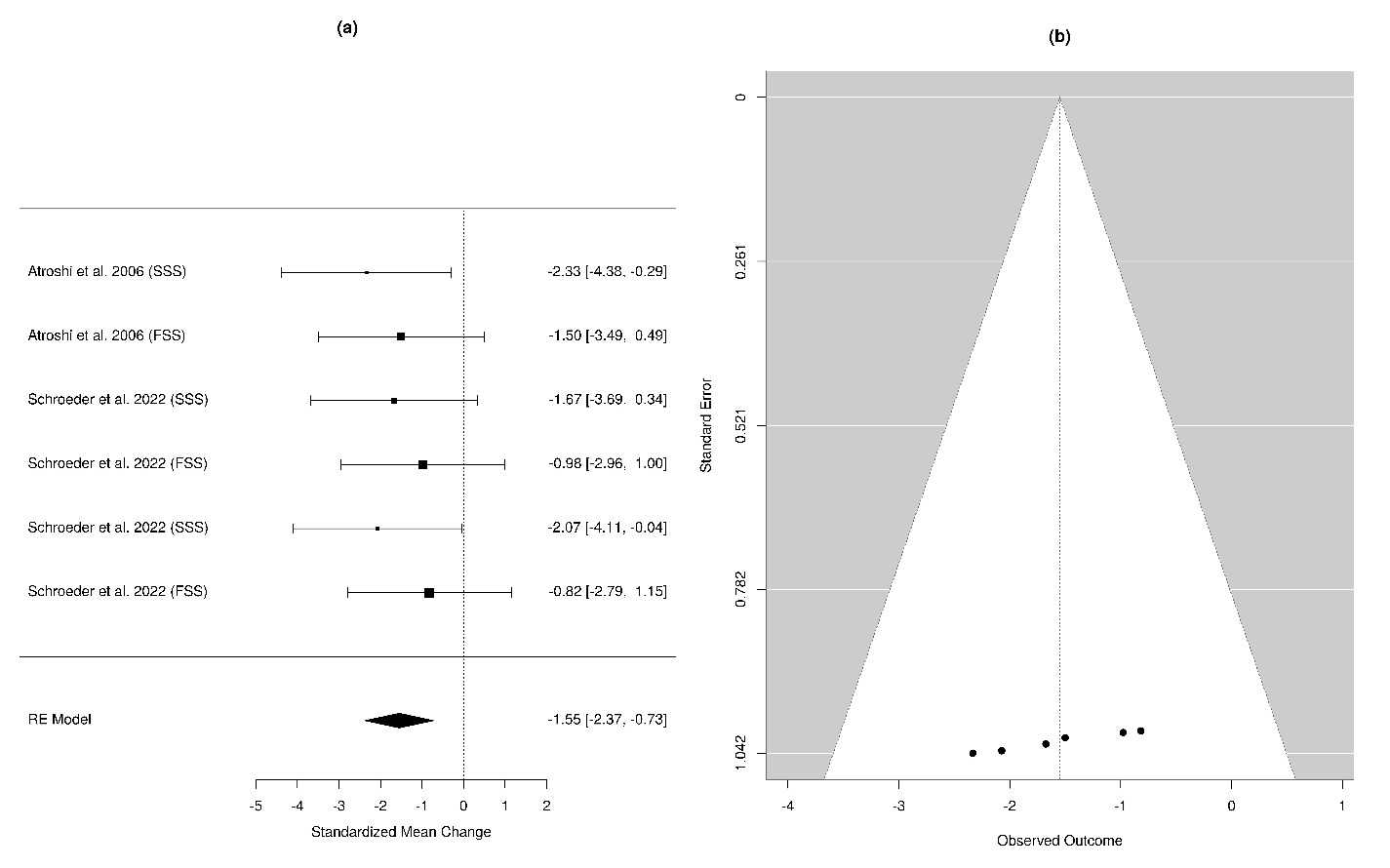
**

Figure S15: A: Standardised mean change for Endoscopic CTR BCTQ score at 6 weeks (I^2^ = 0%). B: funnel plot sensitivity analysis. RE: random effects; FSS: Functional Severity Score; SSS: Symptom Severity Score.

**S16: A: Standardised mean change for Endoscopic CTR BCTQ score at 12 weeks (I^2^ = 0%). B: funnel plot sensitivity analysis.**

**
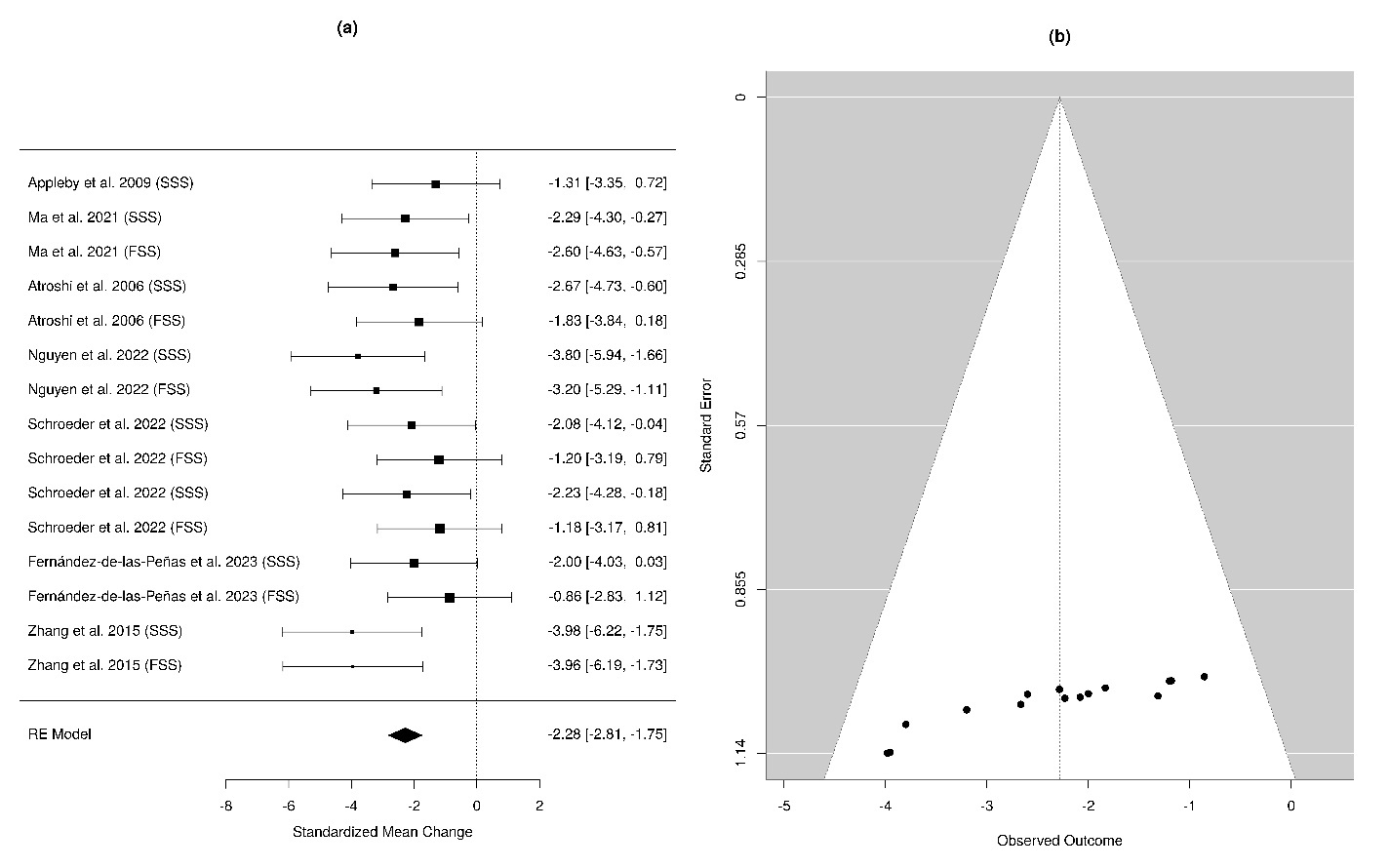
**

Figure S16: A: Standardised mean change for Endoscopic CTR BCTQ score at 12 weeks (I^2^ = 0%). B: funnel plot sensitivity analysis. RE: random effects; FSS: Functional Severity Score; SSS: Symptom Severity Score.

**S17: A: Standardised mean change for Endoscopic CTR BCTQ score at 24 weeks (I^2^ = 31%). B: funnel plot sensitivity analysis.**

**
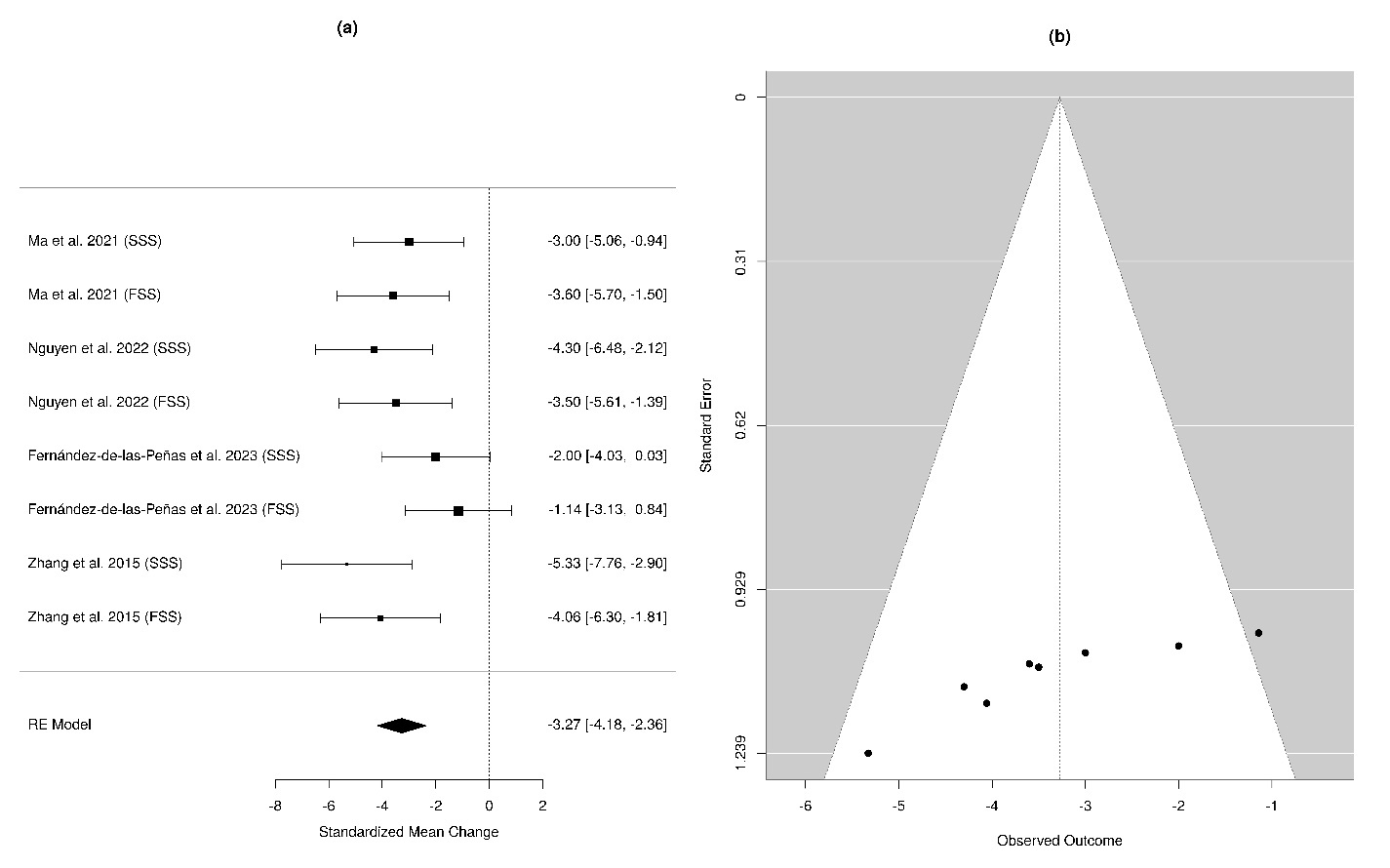
**

Figure S17: A: Standardised mean change for Endoscopic CTR BCTQ score at 24 weeks (I^2^ = 31%). B: funnel plot sensitivity analysis. RE: random effects; FSS: Functional Severity Score; SSS: Symptom Severity Score.

**S18: A: Standardised mean change for Endoscopic CTR BCTQ score at 52 weeks (I^2^ = 95%). B: funnel plot sensitivity analysis.**

**
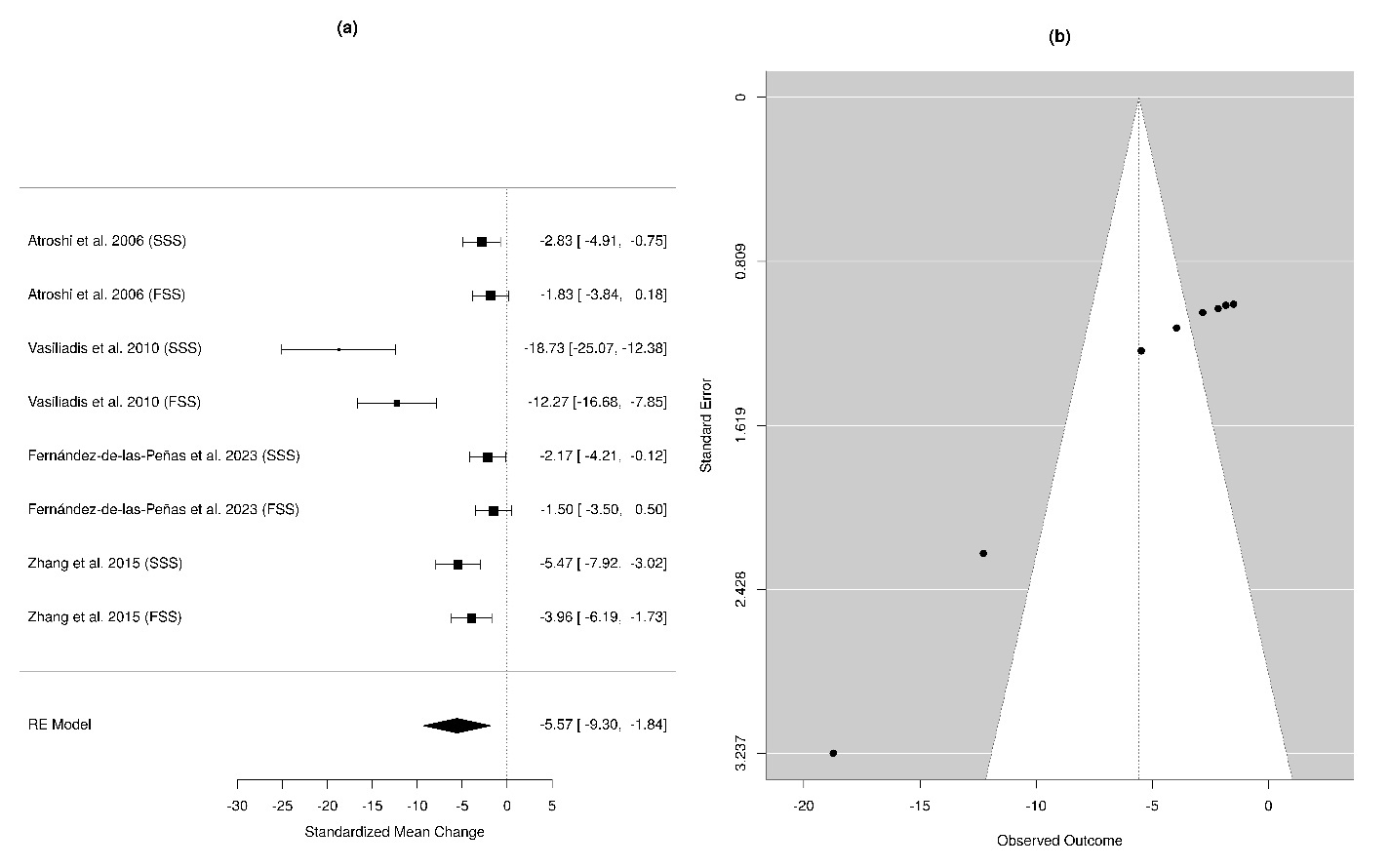
**

Figure S18: A: Standardised mean change for Endoscopic CTR BCTQ score at 52 weeks (I^2^ = 95%). B: funnel plot sensitivity analysis. RE: random effects; FSS: Functional Severity Score; SSS: Symptom Severity Score.

**S19: A: Standardised mean change for Endoscopic CTR BCTQ score at 72 weeks (I^2^ = 0%). B: funnel plot sensitivity analysis.**

**
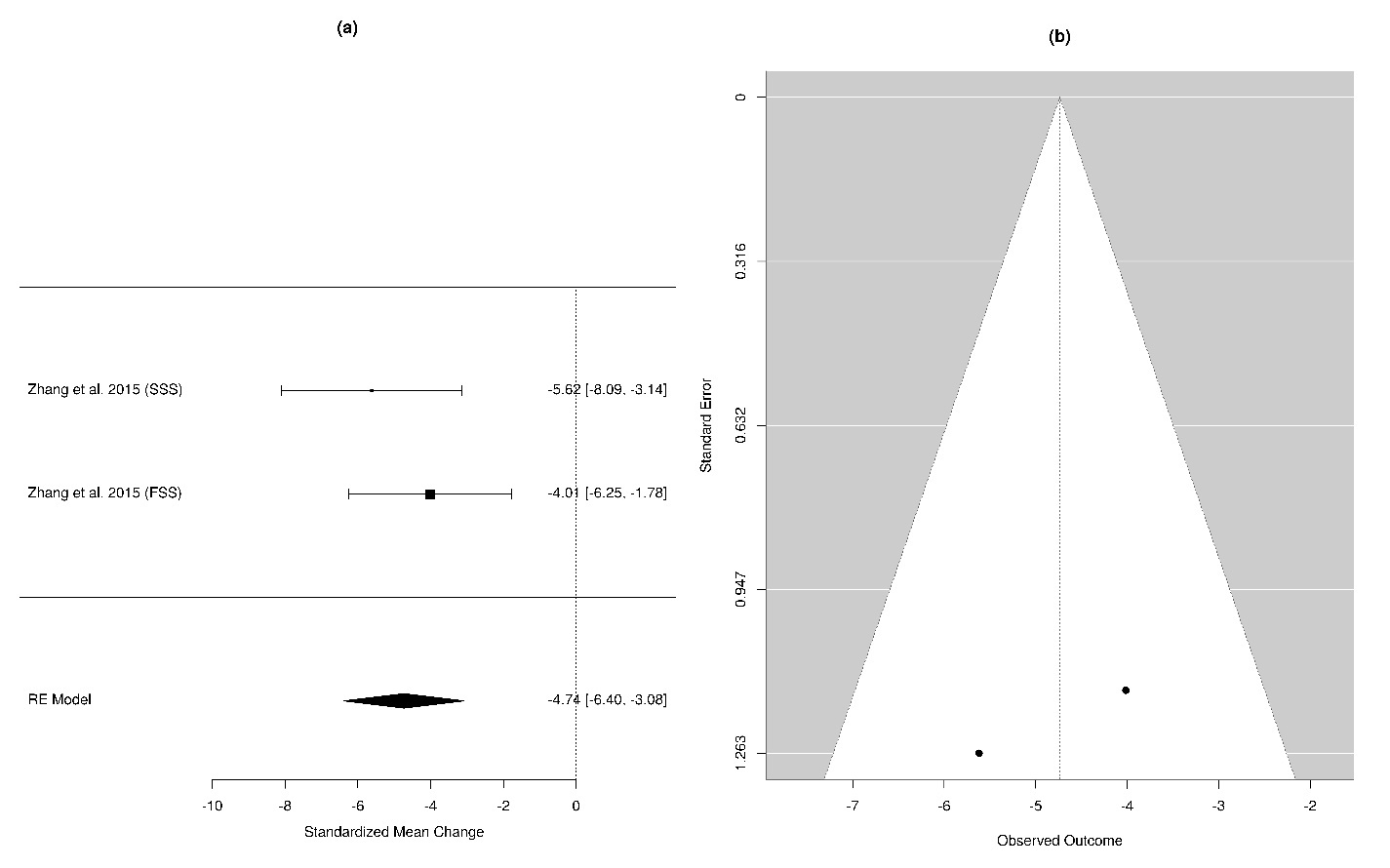
**

Figure S19: A: Standardised mean change for Endoscopic CTR BCTQ score at 72 weeks (I^2^ = 0%). B: funnel plot sensitivity analysis. RE: random effects; FSS: Functional Severity Score; SSS: Symptom Severity Score.

**S20: A: Standardised mean change for Endoscopic CTR BCTQ score at 104 weeks (I^2^ = 0%). B: funnel plot sensitivity analysis.**

**
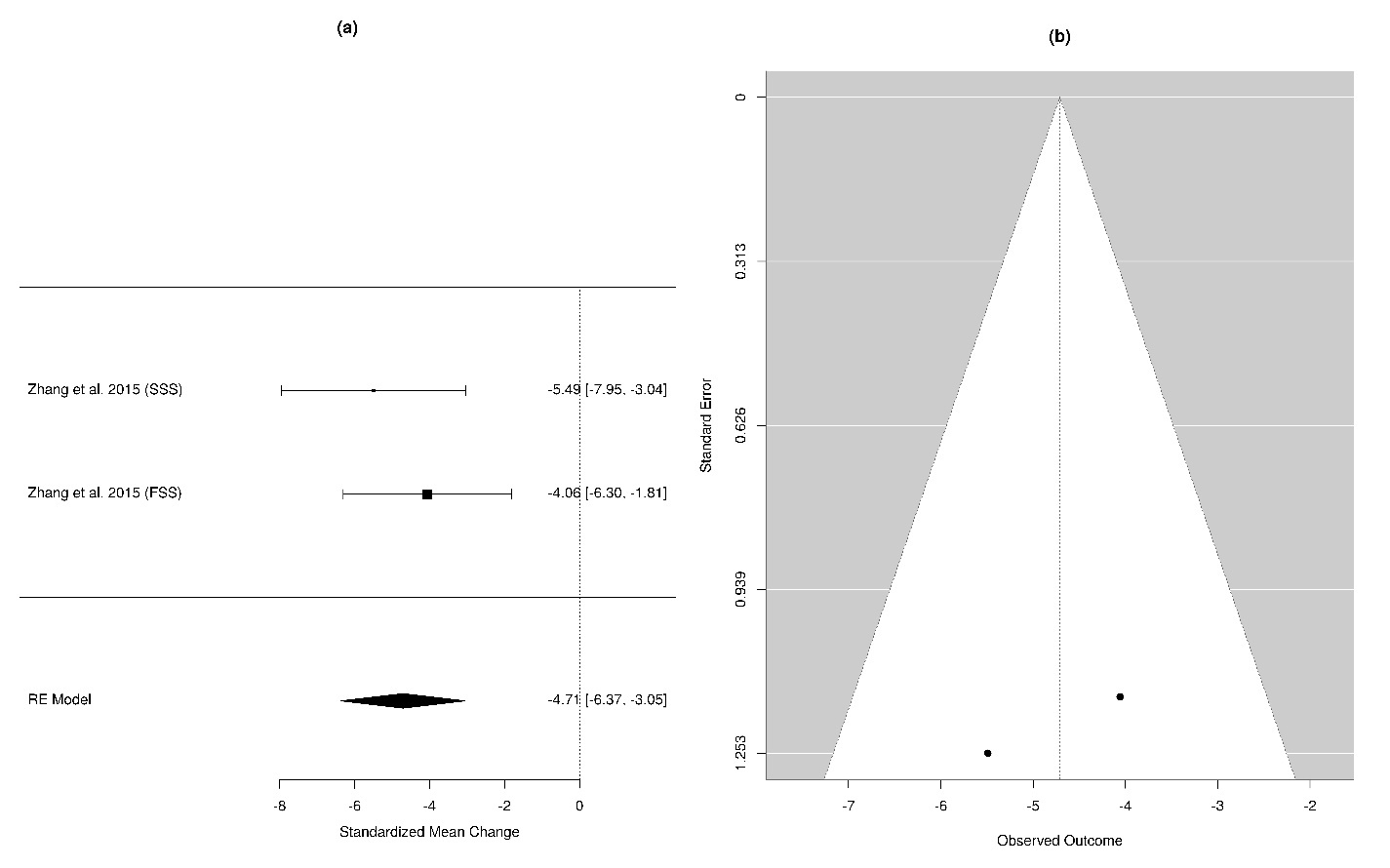
**

Figure S20: A: Standardised mean change for Endoscopic CTR BCTQ score at 104 weeks (I^2^ = 0%). B: funnel plot sensitivity analysis. RE: random effects; FSS: Functional Severity Score; SSS: Symptom Severity Score.

**S21: Change in qDASH score for open and endoscopic carpal tunnel release over time. Each line represents a study arm. A decrease in score over time indicates recovery to a plateau.**


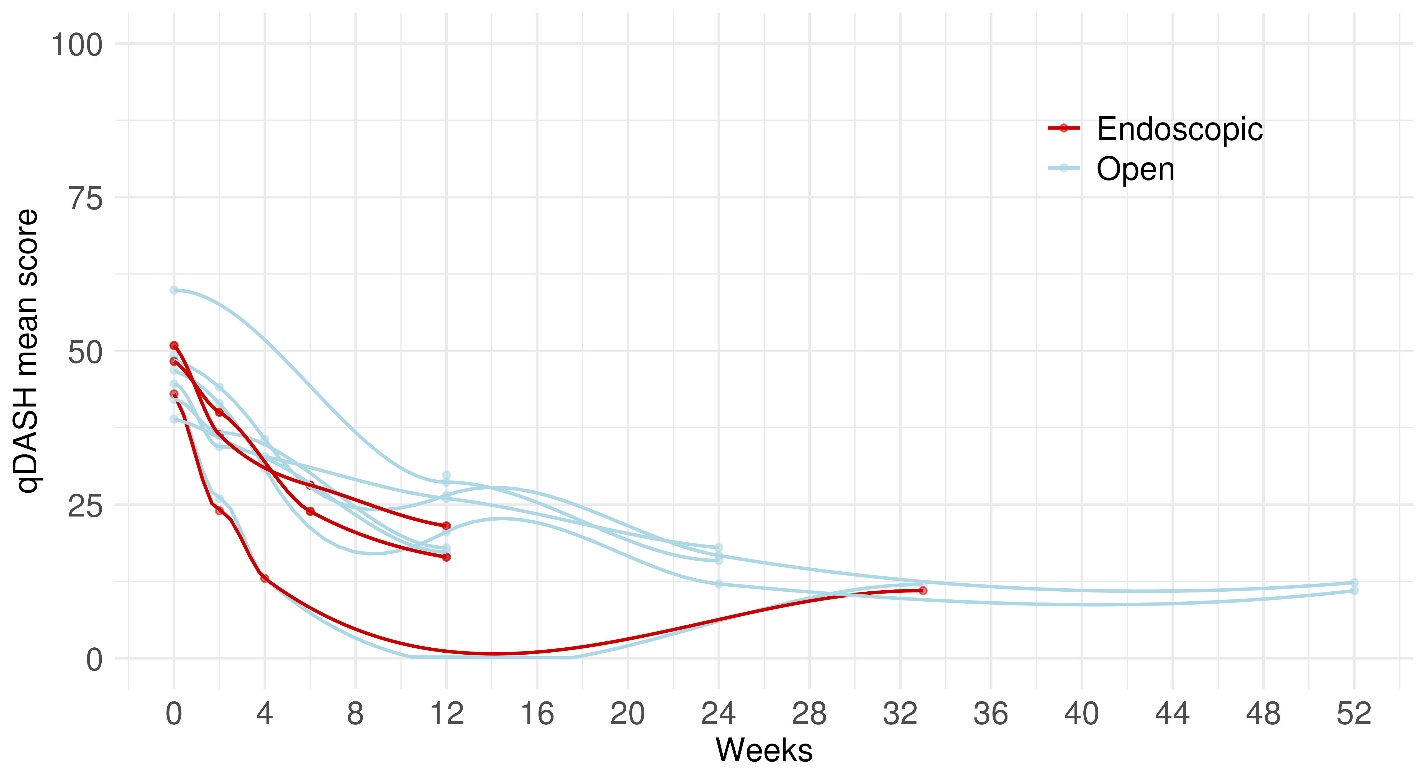


Figure S21: Change in qDASH score for open and endoscopic carpal tunnel release over time. Each line represents a study arm. A decrease in score over time indicates recovery to a plateau.

**Supplementary Table**

| **S1: Summary of the characteristics of included studies.** | | | | | | | |
| --- | --- | --- | --- | --- | --- | --- | --- |
|  | | | | | | | |
| Table S1. Summary of the characteristics of included studies. | | | | | | | |
| Study Author | Study type | Interventions | Sample size | M:F | Age (mean) | Outcome measures | Measurement time-points post operatively (weeks) |
| Sunjic Roguljic et al. 2023 | RCT | Open | 50 | 0.35 | 61.6 | POSAS | 0, 2, 6, 12 |
|  |  | Open | 50 | 0.52 |  |  |  |
| Theopold et al. 2012 | RCT | Open | 18 | 0.38 | 57.9 | POSAS | 0, 6 |
|  |  | Open | 20 | 2.10 | 52.9 |  |  |
| Macfarlane et al. 2014 | Prospective cohort | Open | 25 | 0.39 | 58.6 | Q-DASH | 0, 2, 6 |
|  |  | Open | 28 | 0.33 | 57.7 |  |  |
| Suwannaphisit et al. 2021 | RCT | Open | 71 | 0.13 | 58.1 | BCTQ, POSAS | 0, 2, 6, 12 |
|  |  | Open | 71 | 0.25 | 60.2 |  |  |
| Teng et al. 2019 | Retrospective cohort | Endoscopic | 133 | 0.20 | 41.2 | BCTQ | 0, 4, 12, 24 |
|  |  | Open | 40 | 0.40 | 43.2 |  |  |
|  |  | Open | 38 | 0.90 | 42.7 |  |  |
| Miles et al. 2021 | Retrospective cohort | Endoscopic | 595 | 0.35 | 58 | PROMIS | 0, 2 |
|  |  | Open | 95 | 0.38 |  |  |  |
| Broeke et al. 2019 | Prospective cohort | Open | 72 | 0.36 | ND | BCTQ | 0, 12, 52 |
| Macdermid et al. 2003 | RCT | Open | 91 | 0.47 | 53 | McGill Pain Questionnaire, SF-36 | 0, 1, 6 12 |
|  |  | Endoscopic | 32 | 0.45 | 45 |  |  |
| McMahon et al. 2020 | Prospective cohort | Open | 20 | 0.33 | 57.7 | PROMIS | 0, 2, 4 |
| Shetty et al. 2023 | Prospective cohort | Open + Endoscopic | 405 | 0.49 | 57 | PROMIS | 0, 2 |
|  |  | Open + Endoscopic | 100 | 0.56 |  |  |  |
| Wu et al. 2023 | RCT | Open | 58 | ND | ND | POSAS | 0, 2, 6 |
|  |  | Open | 67 |  |  |  |  |
| Rimdeika et al. 2019 | RCT | Open | 33 | 0.38 | 59 | DASH | 0, 3, 12 |
|  |  | Endoscopic | 71 | 0.27 | 57 |  |  |
| Katz et al. 1994 | Prospective cohort | Open | 104 | 0.43 | 55 | BCTQ | 0, 12 |
|  |  | Endoscopic |  |  |  |  |  |
| Atthakomol et al.2022 | RCT | Open | 11 | 0.00 | 54 | MHQ | 0, 2 |
|  |  | Open | 12 | 0.33 |  |  |  |
| Nabhan et al. 2011 | RCT | Endoscopic | 22 | ND | 55 | MHQ | 0, 2, 24 |
|  |  | Endoscopic | 21 |  |  |  |  |
| Kronlage et al. 2015 | Retrospective cohort | Open | 95 | ND | 60 | CTSS | 0, 2, 4, 8, 12, 52 |
| Zumsteg et al. 2017 | Prospective cohort | Open | 27 | 0.13 | 55 | MHQ, BCTQ | 0, 1, 3, 6, 12 |
| Heybeli et al. 2002 | Prospective cohort | Open | 44 | 0.10 | 48 | BCTQ | 0, 12, 24 |
| Demirci et al. 2002 | Prospective cohort | Steroid | 46 | 0.01 | 45.3 | BCTQ | 0, 12, 24 |
|  |  | Open | 44 | 0.16 | 48.0 |  |  |
| Ucan et al. 2006 | RCT | Splint | 23 | 0.04 | 44.5 | BCTQ | 0, 12, 24 |
|  |  | Splint + steroid | 23 | 0.09 | 44.5 |  |  |
|  |  | Open | 11 | 0.09 | 45.3 |  |  |
| Hu et al. 2022 | Retrospective cohort | Open | 24 | 0.21 | 54.6 | BCTQ, Kellys Grade | 0, 4, 12 |
|  |  | Open | 28 | 0.18 |  |  |  |
| Wang et al. 2022 | Retrospective cohort | Open | 58 | 0.21 | 49.6 | BCTQ, Kellys Grade | 0, 4, 12, 24 |
|  |  | Open | 74 | 0.23 | 47.3 |  |  |
| Appleby et al. 2009 | Prospective cohort | Open | 29 | 0.28 | 48.7 | BCTQ, DASH, SF-36 | 0, 12 |
|  |  | Endoscopic |  |  |  |  |  |
| Fazil et al. 2022 | RCT | Open | 64 | 0.23 | 44.0 | BCTQ | 0, 2, 6, 12, 24, 52 |
|  |  | Open | 58 | 0.29 | 46.0 |  |  |
| Ma et al. 2021 | Prospective cohort | Open | 85 | 0.27 | 48.5 | BCTQ, Kellys Grade | 0, 4, 12, 24 |
|  |  | Endoscopic | 89 | 0.26 | 50.4 |  |  |
| Grandizio et al. 2021 | RCT | Open + Endoscopic | 33 | 0.27 | 56 | Q-DASH, BCTQ, PROMIS | 0, 2 |
|  |  | Open + Endoscopic | 35 | 0.23 | 50 |  |  |
| Atroshi et al. 2006 | RCT | Endoscopic | 63 | 0.30 | 44 | BCTQ, SF-12 | 3 , 6, 12, 52 |
|  |  | Open | 65 | 0.20 | 44 |  |  |
| Nguyen et al. 2022 | Prospective cohort | Endoscopic | 77 | 0.14 | 50.6 | BCTQ | 0, 1, 4, 12, 24 |
| Niedermeier et al. 2020 | Prospective cohort | Endoscopic | 46 | 0.23 | 52.1 | Q-DASH, PSQI | 0, 2, 6 |
|  |  | Open | 15 |  |  |  |  |
| Gaspar et al. 2019 | RCT | Endoscopic | 30 | 0.27 | 49.7 | Q-DASH, ISI and PSQI | 0, 2, 4, 33 |
|  |  | Open | 30 | 0.3 | 49.1 |  |  |
| Zhang et al. 2023 | Prospective cohort | Open | 63 | 0.46 | 59.6 | Q-DASH | 0, 12 |
| Vasiliadis et al. 2010 | Prospective cohort | Endoscopic | 37 | 0.3 | 53.1 | BCTQ, DASH | 0, 1, 2, 52 |
|  |  | Open | 35 | 0.29 | 54.9 |  |  |
| Calzado-Alvarez et al. 2024 | RCT | Z-plasty | 55 | 0.18 | 56.1 | BCTQ | 0, 3, 24 |
|  |  | Open | 54 | 0.35 | 55.5 |  |  |
| Zhang et al. 2015 | RCT | Open + subneural reconstruction | 68 | 0.31 | 45.0 | BCTQ, MHQ | 0, 4, 12, 24, 52, 72, 104 |
|  |  | Open | 92 | 0.36 | 47.0 |  |  |
|  |  | Endoscopic | 53 | 0.30 | 44.0 |  |  |
| Padua et al. 2003 | RCT | Open | 10 | 0.1 | 54.1 | BCTQ | 0, 2, 12 |
|  |  | Open + steroid | 10 | 0.1 | 53.2 |  |  |
| Fucs et al. 2023 | RCT | Open | 28 | 0.36 | ND | Q-DASH | 0, 4, 12, 24 |
|  |  | Z-plasty | 28 | 0.36 | ND |  |  |
| Yagci et al. 2006 | RCT | Splint | 23 | 0.05 | 44.5 | BCTQ | 0, 12 |
|  |  | Splint + steroid | 23 | 0.05 | 44.5 |  |  |
|  |  | Open | 11 | 0.1 | 45.3 |  |  |
| Schroeder et al. 2024 | RCT | Endoscopic | 50 | 0.32 | 56 | Q-DASH, BCTQ | 0, 2, 6, 12 |
|  |  | Endoscopic | 55 | 0.34 | 59 |  |  |
| Fernández-de-las-Peñas et al. 2023 | RCT | Percutaneous | 35 | 0 | 46 | BCTQ | 0, 4, 12, 24, 52 |
|  |  | Endoscopic | 35 | 0 | 47 |  |  |
| Malisorn 2023 | Retrospective cohort | Open | 60 | 0.28 | 55.36 | BCTQ, Q-DASH | 0, 2, 4 |
|  |  | Open | 60 | 0.28 | 55.48 |  |  |
| Bassil et al. 2024 | Prospective cohort | Open | 221 | 0.23 | 58.1 | BCTQ | 0, 4, 12, 24 |
|  |  | Open | 194 | 0.30 | 58.8 |  |  |
| Roguljic et al. 2024 | RCT | Open | 50 | 0.35 | 60.1 | ISI, PSQI, SF-36 | 0, 2, 6, 24, 52 |
|  |  | Open | 48 | 0.52 | 63.0 |  |  |
| Razavipour et al. 2021 | Prospective cohort | Open | 30 | NR | NR | Q-DASH, BCTQ | 0, 12, 24 |
| Shazad et al. 2022 | Prospective cohort | Open | 32 | NR | 45.44 | GSS | 0, 12, 24, 52 |
| Castro-Menéndez et al. 2023 | Prospective cohort | Open | 25 | 0.32 | 49.2 | BCTQ | 0, 6, 12 |
| Zyluk & Szlosser, 2013 | RCT | Open | 45 | 0.01 | 61 | BCTQ | 0, 4, 24 |
|  |  | Open | 48 | 0.13 | 55 |  |  |
| Ergen et al. 2024 | RCT | Open | 15 | 0.07 | 54.47 | PSFS, BCTQ (SSS) | 0, 6, 12 |
|  |  | Open | 15 | 0.15 | 53.73 |  |  |
| Flecke et al. 2018 | RCT | Open | 24 | 0.41 | 62.2 | MHQ, DASH | 0, 3 |
|  |  | Open | 22 | 0.5 | 63.8 |  |  |
| Tulipan et al. 2017 | Prospective cohort | Open | 81 | 0.46 | 62.3 | BCTQ, Q-DASH | 0, 2 12 |
|  |  | Open | 149 | 0.39 | 61.7 |  |  |
| RCT: Randomized controlled trial; POSAS: patient observer scar assessment score; Q-DASH: quick Disabilities of the Arm, Shoulder and Hand; BCTQ: Boston Carpal Tunnel Questionnaire; PROMIS: Patient-Reported Outcomes Measurement Information System; ND: not discussed; SF-36: Short-Form 36; DASH: Disabilities of the Arm, Shoulder and Hand; MHQ: Michigan Hand Outcomes Questionnaire; CTSS: Carpal tunnel severity score; SF-12: Short-Form 12; PSQI: Pittsburgh Sleep Quality Index; ISI: Insomnia Severity Index; GSS: Global symptom score; PSFS: Patient Specific Functional Scale. | | | | | | | |

**S2: Risk of bias assessment results of all included studies**

| Table S2: Risk of bias assessment of all included studies. | | | | | | | | | |  |
| --- | --- | --- | --- | --- | --- | --- | --- | --- | --- | --- |
| Study | Q1 | Q2 | Q3 | Q4 | Q5 | Q6 | Q7 | Q8 | Q9 |  |
| Sunjic Roguljic et al. 2023 | + | + | + | - | + | + | + | + | + |  |
| Theopold et al. 2012 | + | + | + | + | - | + | + | + | + |  |
| Macfarlane et al. 2014 | + | + | + | + | - | + | + | - | + |  |
| Suwannaphisit et al. 2021 | + | + | + | - | + | + | + | + | + |  |
| Teng et al. 2019 | + | + | - | ? | + | + | + | - | ? |  |
| Miles et al. 2021 | + | + | + | + | + | + | + | - | + |  |
| Broeke et al. 201949 | + | + | + | - | - | + | + | - | - |  |
| Nazerani et al. 201450 | + | + | + | + | + | + | + | - | + |  |
| Macdermid et al. 2003 | + | + | + | + | + | + | + | + | + |  |
| McMahon et al. 2020 | + | + | + | + | - | + | + | + | + |  |
| Shetty et al. 2023 | + | + | + | - | + | - | + | - | ? |  |
| Wu et al. 2023 | + | + | ? | - | + | + | + | - | - |  |
| Rimdeika et al. 2019 | + | + | + | + | + | + | + | - | + |  |
| Katz et al. 1994 | + | + | + | + | + | - | + | + | - |  |
| Atthakomol et al.2022 | + | + | + | + | - | + | + | + | + |  |
| Nabhan et al. 2011 | + | + | + | + | - | + | + | - | + |  |
| Kronlage et al. 2015 | + | + | + | + | - | + | + | - | + |  |
| Zumsteg et al. 2017 | + | + | + | - | - | + | + | - | - |  |
| Heybeli et al. 2002 | + | + | + | + | - | - | + | - | + |  |
| Demirci et al. 2002 | + | + | + | + | - | + | + | - | + |  |
| Ucan et al. 2006 | + | + | + | + | - | + | + | - | + |  |
| Hu et al. 2022 | - | + | + | ? | - | + | + | - | + |  |
| Wang et al. 2022 | + | + | + | + | + | + | + | - | + |  |
| Appleby et al. 2009 | + | + | + | ? | - | + | + | - | + |  |
| Fazil et al. 2022 | + | + | - | + | + | + | + | + | + |  |
| Ma et al. 2021 | + | + | + | + | + | + | + | - | + |  |
| Grandizio et al. 2021 | + | + | + | + | - | + | + | - | + |  |
| Atroshi et al. 2006 | + | + | + | - | + | + | + | + | + |  |
| Nguyen et al. 2022 | + | + | + | + | - | + | + | - | + |  |
| Niedermeier et al. 2018 | + | + | + | + | - | - | + | - | - |  |
| Gaspar et al. 2019 | + | + | + | + | - | + | + | - | + |  |
| Zhang et al. 2023 | + | + | + | + | - | + | + | - | - |  |
| Tulipan et al. 2017 | + | + | + | + | + | + | + | - | - |  |
| Bassil et al. 2024 | + | + | + | + | + | + | + | ? | + |  |
| Padua et al. 2003 | + | + | + | + | + | + | + | + | + |  |
| Schroeder et al. 2024 | + | + | + | + | + | + | + | ? | - |  |
| Fucs et al. 2023 | + | + | - | ? | + | - | + | ? | ? |  |
| Roguljic et al. 2024 | + | + | + | + | + | + | + | ? | + |  |
| Malisorn 2023 | + | + | + | + | + | + | + | ? | ? |  |
| Razavipour et al. 2021 | + | + | - | + | ? | + | + | ? | - |  |
| Fernández-de-las-Peñas et al. 2023 | + | + | - | + | + | + | + | ? | + |  |
| Zhang et al. 2015 | + | + | + | + | + | + | + | ? | ? |  |
| Vasiliadis et al. 2010 | + | + | + | + | + | + | + | - | + |  |
| Calzado-Alvarez et al. 2024 | + | + | + | + | + | + | + | + | + |  |
| Yagci et al. 2006 | + | + | + | + | - | ? | + | - | + |  |
| Castro-Menéndez et al. 2023 | + | + | + | + | + | + | + | ? | + |  |
| Zyluk & Szlosser, 2013 | + | + | + | + | + | ? | + | + | + |  |
| Ergen et al. 2024 | + | + | + | + | + | + | + | + | + |  |
| Flecke et al. 2018 | + | + | + | + | + | ? | + | - | + |  |
| Red represents high risk of bias; Amber represents moderate risk of bias and green represents low risk of bias.    Symbols “+” represents “yes”, “-“ represents “no” and “?” represents “cannot determine”. | | | | | | | | | |  |
|  |  |  |  |  |  |  |  |  |  |  |

**Table S3: Cumulative Standardised mean change (SMC) in Boston Carpal Tunnel Questionnaire scores from baseline for open and endoscopic carpal tunnel release (CTR) at 1, 2, 3, 4, 6, 12, 24, 52, 72 AND 104-week timepoints.** 95% Confidence intervals are presented in brackets. A negative value represents an improvement from baseline. All values have been rounded to two decimal places.

| Weeks | Open CTR | | Endoscopic CTR | |
| --- | --- | --- | --- | --- |
|  | SMC | Cumulative SMC | SMC | Cumulative SMC |
| 1 | 10.29 (6.35, 14.21) | 1.69 (1.39, 1.99) | -2.83 (-7.80, 2.14) | -0.74 (-1.23, -0.25) |
| 2 | -2.22 (-4.54, 0.10) | -1.38 (-1.82, -0.95) | -3.71 (-7.61, 0.19) | -2.41 (-3.10, -1.71) |
| 3 | -1.30 (-2.3, -0.3) | -5.87 (-6.48, -5.26) | -1.39 (-2.86, 0.08) | -2.71 (-3.56, -1.87) |
| 4 | -1.96 (-2.38, -1.54) | -5.50 (-6.17, -4.83) | -2.02 (-2.80, -1.24) | -2.72 (-3.70, -1.75) |
| 6 | -4.19 (-6.66, -1.73) | -5.72 (-6.45, -4.99) | -1.55 (-2.37, -0.73) | -2.96 (-4.04, -1.88) |
| 12 | -2.73 (-3.39, -2.07) | -6.79 (-7.57, -6.00) | -2.28 (-2.81, -1.75) | -3.35 (-4.53, -2.17) |
| 24 | -3.98 (-5.22, -2.74) | -7.52 (-8.35, -6.69) | -3.27 (-4.18, -2.36) | -3.99 (-5.26, -2.71) |
| 52 | -9.29 (-13.49, -5.10) | -8.58 (-9.46, -7.70) | -5.57 (-9.30, -1.84) | -3.88 (-5.24, -2.52) |
| 72 | -4.13 (-6.74, -1.52) | -10.41 (-11.38, -9.43) | -4.74 (-6.40, -3.08) | NA |
| 104 | -4.04 (-6.75, -1.36) | -10.32 (-12.02, -8.62) | -4.71 (-6.37, -3.05) | -3.82 (-5.76, -1.88) |
